# Identifying Novel Actionable Targets in Colon Cancer

**DOI:** 10.3390/biomedicines9050579

**Published:** 2021-05-20

**Authors:** Maria Grazia Cerrito, Emanuela Grassilli

**Affiliations:** Department of Medicine and Surgery, University of Milano-Bicocca, Via Cadore 48, 20900 Monza, Italy; mariagrazia.cerrito@unimib.it

**Keywords:** colon cancer, drug resistance, target therapy, high-throughput screen, si/sh-RNA screen, CRISPR/Cas9 knockout screen, drug re-purposing, drug re-positioning

## Abstract

Colorectal cancer is the fourth cause of death from cancer worldwide, mainly due to the high incidence of drug-resistance toward classic chemotherapeutic and newly targeted drugs. In the last decade or so, the development of novel high-throughput approaches, both genome-wide and chemical, allowed the identification of novel actionable targets and the development of the relative specific inhibitors to be used either to re-sensitize drug-resistant tumors (in combination with chemotherapy) or to be synthetic lethal for tumors with specific oncogenic mutations. Finally, high-throughput screening using FDA-approved libraries of “known” drugs uncovered new therapeutic applications of drugs (used alone or in combination) that have been in the clinic for decades for treating non-cancerous diseases (re-positioning or re-purposing approach). Thus, several novel actionable targets have been identified and some of them are already being tested in clinical trials, indicating that high-throughput approaches, especially those involving drug re-positioning, may lead in a near future to significant improvement of the therapy for colon cancer patients, especially in the context of a personalized approach, i.e., in defined subgroups of patients whose tumors carry certain mutations.

## 1. Introduction

### 1.1. Biology of Colorectal Cancer

**Figure 1:**
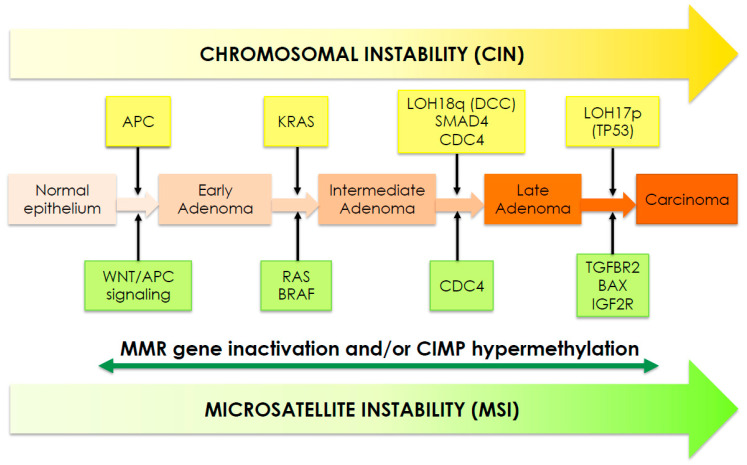
Schematic representation of CRC progression along the three different pathways according to the Fearon and Vogelstein model. CIN, chromosomal instability; MSI, microsatellite instability; CIMP, CpG island methylator; MMR, DNA mismatch repair; LOH, loss of heterozygosity. Independently of the pathway, a defect in the APC/beta-catenin axis marks the onset of the transformation process from normal epithelia to early adenoma. A defect along the KRAS/BRAF pathway is required to progress to intermediate adenoma. Loss or silencing of different tumor suppressor genes finally determines the progression to late adenoma and then to carcinoma. In the CIN pathway, the transition to the carcinoma stage is marked by the inactivation of the tumor-suppressor gene TP53, whose product is pivotal in regulating DNA repair, cell cycle arrest, senescence, apoptosis and metabolism in response to a variety of stress signals. Therefore, its loss contributes to drug resistance and to the propagation of damaged DNA to daughter cells, increasing the mutational load. TP53 mutation or loss of it has been reported in 50–75% of CRC cases and it is associated with the progression and outcome of sporadic CRC [2,3].

Colorectal cancer (CRC) formation begins with the transformation of a normal colorectal epithelium to a benign adenoma. It then progresses through the stepwise accumulation of multiple genetic and epigenetic aberrations by three major pathways: chromosomal instability (CIN), microsatellite instability (MSI) and CpG island methylator (CIMP+) phenotype. These pathways are not mutually exclusive, with some tumors exhibiting features of multiple pathways; in all cases, the point of arrival is the carcinoma which subsequently progresses to an invasive and metastatic tumor [1] (Figure 1).

**Figure 2:**
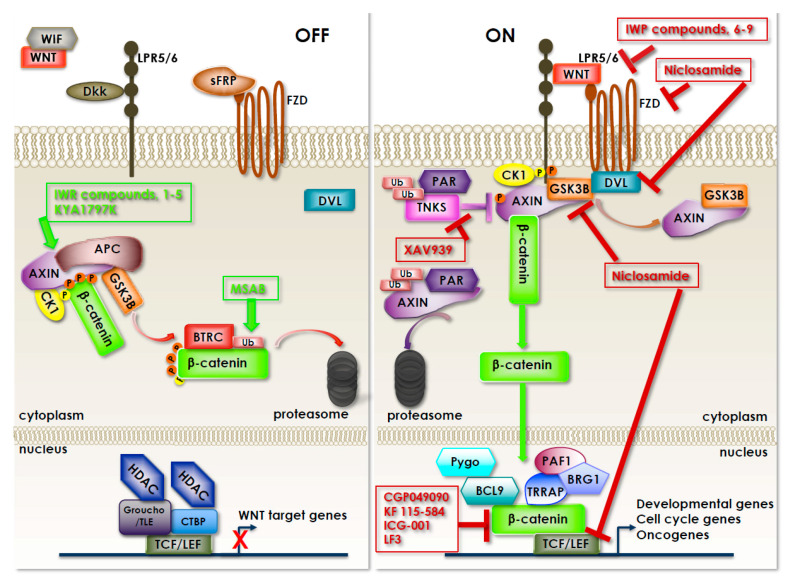
WNT/beta-catenin canonical signaling pathway in CRC and identified inhibitors. When WNT proteins are sequestered by WNT inhibitory factor-1 (WIF-1), the member of the frizzled (FZD) family of atypical G protein-coupled receptors is inhibited by a secreted frizzled-related protein (SFRP) and the co-receptor lipoprotein receptor-related protein (LPR) 5 or 6 is bound to Dickkopf (DKK); WNT signaling is therefore off. As a consequence, the receptor complex is not formed and the destruction complex is assembled in the cytoplasm, where APC and AXIN serve as a scaffold to recruit CK1 and GSK3B, both of which phosphorylate beta-catenin, thus targeting it for BTRC-mediated ubiquitination and subsequent proteasome-mediated degradation. In the nucleus, TCF/LEF transcription factor sits on the promoter of WNT-regulated genes where, via binding a member of the Groucho/TLE family of transcription repressors or CtBP, it recruits HDAC to repress transcription of the downstream genes. The signaling starts when WNT is freed and can bind a member of FZD family LPR5/6, thus forming the receptor complex which, via the binding of the adaptor protein Disheveled (DVL), recruits to the membrane the destruction complex, disrupting it. Tankyrases (TNKSs,) by poly-ADP-ribosylating AXIN, prime it for ubiquitination and subsequent proteasome-mediated degradation. Alternatively, AXIN can sequester GSK3B away from the complex; in both ways beta-catenin is released from the destruction complex and translocates to the nucleus, where it displaces transcription repressors and complexes with TCF/LEF to recruit several transcriptional coactivators (Pygo, BCL9) and histone modifiers (such as TRRAP, PAF1, BRG1, etc.) in order to promote the transcription of the downstream target genes. In the red and green boxes, chemical and re-purposed drugs are identified in the screens described in the text. CGP049090, KF 115-584, ICG-001, LF3: compounds identified as able to displace the interaction of beta-catenin with TCF/LEF transcription factors or recruited coactivators; XAV939: TNKSs inhibitor; IWR compounds, 1–5, KYA1797K: axin stabilizers; IWP compounds, 6–9: inhibitors of WNT production; MSAB: stimulators of beta-catenin ubiquitination [4,5,6,7,8,9].

**Figure 3:**
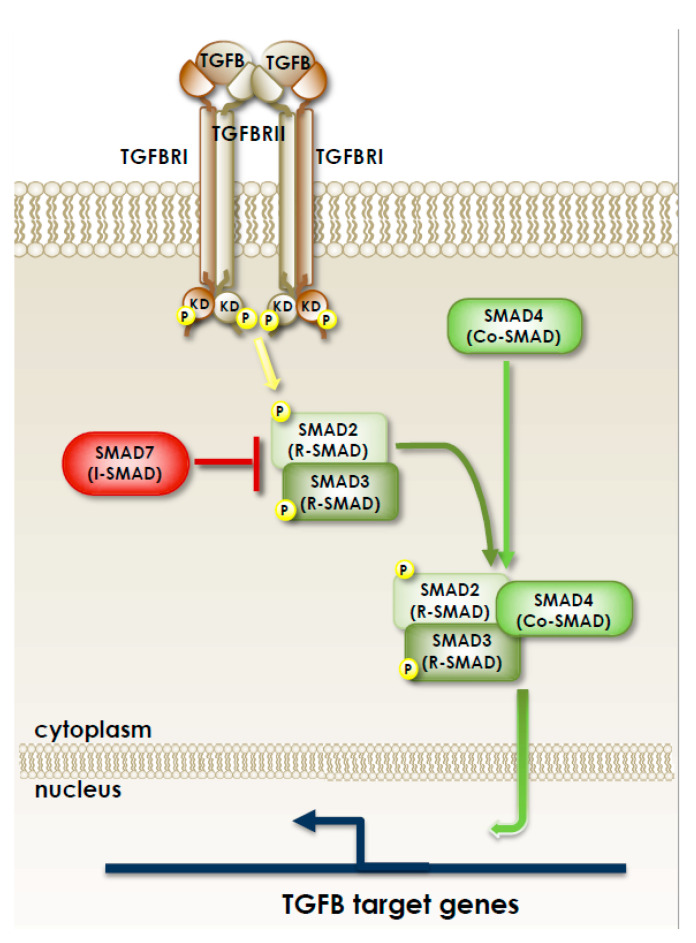
Signaling pathway activated downstream of the TGFBR in CRC. Upon binding the TGFB1 dimer, TGF-beta receptor type-2 (TGFBR2) promotes its dimerization with TGFBR1, resulting in transphosphorylation of TGFBR1. Activated TGFBR1 phosphorylates and activates receptor-regulated SMADs R-SMADs, SMAD2 and SMAD3, thus promoting the trimerization with a co-SMAD (SMAD4). SMAD7 is an inhibitory SMAD (I-SMAD) that can bind to TGFBR1 competing with SMAD2/3 for the catalytic site of phosphorylation, thus preventing the phosphorylation of SMAD2/3. In addition, SMAD7 can promote dephosphorylation/inactivation of TGFBR1 or boost ubiquitination and proteasome-mediated degradation of TGFBR1. Activated SMAD complex enters the nucleus, where it binds DNA directly or indirectly, via other transcription factors, and regulates gene expression, both positively and negatively. SMAD4 inactivation has been reported to correlate with CRC tumor progression, development and distant metastasis. Moreover, its reduced expression or loss was associated with poor survival and prognosis in patients with CRC. In addition, loss of SMAD4 in CRC patients conferred resistance to chemotherapy drugs, such as 5-fluorouracil (5-FU) [10]. Loss-of-function mutations have been found in approximately 10–35% of patients with CRC [11]. Moreover, some studies reported absent, or reduced SMAD4 expression in 66% of CRC samples from patients analyzed [10,11,12,13,14,15,16].

Almost 85% of sporadic CRCs present CIN, which results from defects in chromosomal segregation, telomere stability and the DNA damage response, and leads to gain/losses of chromosomal segments, chromosomal rearrangements and LOH of tumor suppressor genes, such as APC, TP53, DCC and SMAD family members (SMAD2 and SMAD4), eventually resulting in the dysregulation of several important signaling pathways (Figure 2 and Figure 3).

**Figure 4:**
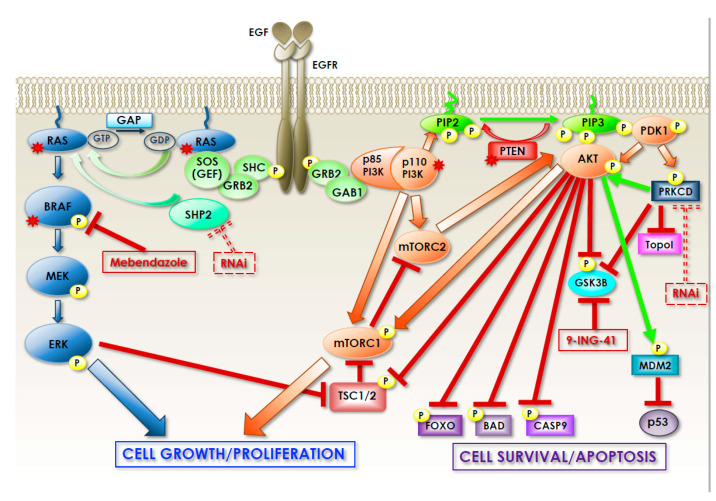
Signaling pathways activated downstream of the EGFR in CRC. Upon binding of the EGF with its dimerized receptors, and subsequent activation by autophosphorylation at multiple C-terminal Tyr residues, several proteins can be recruited to trigger different signaling pathways. For clarity, only two major pathways mainly affected by mutational events in CRC are presented in the figure. The phosphorylated C-terminal domain binds SHC and GRB2, which in turn recruits SOS to initiate ERK/MAPK signaling. SOS is a GDP Exchange Factor (GEF) that catalyzes the conversion of GDP to GTP of RAS, activating it. Active RAS recruits BRAF, which is activated by dephosphorylation and phosphorylation events. Activated BRAF phosphorylates and activates MEK1/2, which in turn activates ERK1/2. Phospho-ERK1/2 have various cytoplasmic and nuclear targets, which aid in the transcription and translation of cell cycle and cell growth-related genes. On the other hand, the receptor-bound GRB2 can also bind GAB1 which recruits the p85 regulatory subunit of PI3K that, via binding of the p110 catalytic subunit, (PI3KCA) activates it. PI3KCA-activating mutations occur in approximately 10–20% of CRCs, most of them exhibiting also a KRAS mutation [1]. On the other hand, phosphatase and tensin homolog deleted on chromosome ten (PTEN), by dephosphorylating PIP3, counteracts the PI3K/AKT signaling cascade. Loss of PTEN expression resulting from both genetic (genomic mutations and decreased gene copy numbers) and epigenetic mechanisms (promoter hypermethylation) occurs in 34.5% of cases [21]. Activated PI3K phosphorylates membrane-bound PIP_2_ to PIP_3_, which in turn recruits AKT and PDK1, the latter being responsible for AKT phosphorylation and activation. PDK1 can also phosphorylate protein kinase C delta type (PRKCD) which in turn can activate AKT. In addition, PRKCD can inhibit, by phosphorylation, GSK3B. Active AKT has many substrates, and most of them are inhibited upon phosphorylation (such as pro-apoptotic proteins FOXO, CASP9 and BAD) whereas MDM2, the Ub-ligase targeting p53 for degradation, is activated. Finally, AKT can activate mTOR complex 1 (mTORC1) either via phosphorylating TSC2—thus relieving its inhibitory activity on mTORC1 (via Rheb)—or via directly phosphorylating mTORC1 itself. TSC2-mediated inhibition can also be relieved by ERK-mediated phosphorylation downstream of RAS; finally, mTORC1 can also be activated directly by PI3K-mediated phosphorylation. As a consequence of mTORC1 activation, eIF4E-mediated translation of several proteins involved in cell cycle regulation and cell growth is triggered. Besides mTORC1, PI3K can also directly activate mTOR complex 2 (mTORC2), which in turn amplifies AKT-mediated downstream signaling; excessive signaling is, however, kept in check by a negative feedback loop where mTORC1 inhibits mTORC2. Red stars indicate the occurrence of mutations in the specific protein, all of which are activating but for PTEN, for which a loss of function, either genetic or epigenetic, occurs. Inhibitory drugs identified by the re-purposing of chemical screens described in the text are indicated in the red boxes; hatched red boxes indicate target inhibition identified by genetic screens.

In addition, CIN tumors accumulate mutations in specific oncogenes, KRAS and BRAF being the most affected [17,18]. KRAS mutations are detected in 30–50% of CRCs resulting in constitutive activation of the RAS–RAF–MEK–ERK pathway, downstream and independently of the EGFR (Figure 4). BRAF mutation occurs in approximately 8–10% of CRC patients; being part of the same pathway, BRAF and KRAS mutations tend to be mutually exclusive. Additionally, BRAF mutation leads to sustained MAPK signaling and is associated with poor survival and drug resistance [19,20].

MSI occurs in 15–20% of sporadic CRCs and it is characterized by the high frequency of insertion/deletions of nucleotides in microsatellite DNA repeat sequences due to a malfunctioning of the DNA mismatch repair (MMR) system, which in turn derives from an epigenetic silencing or a germline mutation in one of the MMR genes [3]. Loss/insufficiency of MMR activity leads to replication errors with an increased mutation rate and a higher potential for malignancy. The detection of instability is identified via a PCR-based assay categorizing tumors as either MSI-high (MSI-H), MSI-low (MSI-L) or microsatellite stable (MSS), based on the number of microsatellite markers demonstrating instability [22].

CIMP has been identified in 17% of CRCs and is characterized by epigenetic alterations such as promoter methylation, which results in gene silencing, thus providing an alternative mechanism for loss-of-function of tumor suppressor genes. CIMP status is determined by assessing hypermethylation of the CACNA1G, MLH1, NEUROG1, RUNX3 and SOCS1 promoters [23]. Promoter methylation of tumor suppressor candidate 3 (TUSC3) has been associated with increased EGFR signaling and poor survival rate [24]. CIMP+ CRCs usually are characterized by MSI status (80%), BRAF mutation (53%) and wild-type KRAS [3]. Several other epigenetic aberrations including DNA methylation, histone modifications and chromatin remodeling have been observed and reported to be associated with CRC initiation and progression [25]. In addition, experimental data strongly suggest that epigenetic modifications can also contribute to the resistance to anti-cancer therapies [24]. In the last decade, extensive studies have shown that epigenetic alterations may occur not only at the DNA also at the RNA level, thus leading to crucial modifications in gene expression and pathway activation. In particular, dysregulation of RNA methylation (due to altered expression of specific enzymes) has been linked to the hyperactivation of the MAPK/ERK and the WNT/beta-catenin pathways in the absence of mutations of the main players of the pathway itself [26]. Finally, aberrant epigenetic regulation has been reported to occur via altered expression of members of the long non-coding RNAs (lncRNAs) family. In fact, some nuclear lncRNAs have been demonstrated to regulate gene expression in cis or trans via binding to specific genomic loci, either near to or distant from their transcription sites, and recruiting epigenetic factors, including the DNA methyltransferase and histone modification complex [27].

Lately, the advent of new high-throughput “omics” technologies has added a layer of complexity in the characterization of CRC biology. Besides the evolution in the understanding of its genetic heterogeneity, the use of transcriptomics has identified clearly distinct subtypes of CRC, indicated as consensus molecular subtypes (CMS). CMS determination seems to be critical not only for tumor classification but also for prognostic outcomes and therapeutic decisions, since it encompasses tumor, stromal and immune components and classical histopathological classification. Each CMS is identified by a specific expression profile and pathway activation: CMS1 (microsatellite instability immune), CMS2 (canonical), CMS3 (metabolic), CMS4 (mesenchymal) and a mixed features phenotype representing transitional or intratumoral heterogeneity [28,29]. So far, gene expression analysis has been used to determine CMS; however, recently five immunohistochemistry-based classifiers, CDX2, FRMD6, HTR2B, ZEB1 and KER, have been identified that demonstrate 87% concordance with traditional transcriptome-based classification. The recent classification of four CMS may therefore form the basis for future clinical stratification of CRC with subtype-based targeted interventions [30].

### 1.2. Therapy and Drug Resistance

The treatment of both early-stage and metastatic cancer patients is mainly based on chemotherapy, being the standard approach represented by surgery combined with radiotherapy and/or chemotherapy (depending on tumor site and progression of disease) [28,29,30]. Fluoropyrimidines (such as 5-fluorouracil, 5-FU), oxaliplatin and irinotecan represent the chemotherapy backbones for treating metastatic CRC, and their sequential administration allows median overall survival ranging from 18 to 20 months [31]. However, recurrence after chemotherapy is the barrier to effective clinical outcomes for CRC patients; we reported that, as a mechanism of resistance, 5-FU activates the TGFB pathway [32], and that targeting TGFBRI restored the sensitivity of drug-resistant cells to 5-FU toxicity [33]. In addition, it has been recently demonstrated that a possible reason for tumor regrowth after chemotherapy might be a p53-mediated activation of the WNT/beta-catenin pathway in cells that escape the cytotoxic effect of chemotherapy. Through this regulation, 5-FU induces activation and enrichment of cancer stem cells (CSC) in the residual tumors, contributing to recurrence after treatment. Accordingly, combinatorial treatment with a WNT inhibitor and 5-FU effectively suppresses the CSCs and reduces tumor regrowth after discontinuing the treatment [34].

The CSC hypothesis supports a model where a small population of stem cells drive tumor growth and metastasis and may even predict disease relapse [35]. CSC are inherently more resistant than “more mature” tumor cells to chemotherapeutic drugs and may even enter a quiescent state, resistant to anti-proliferative drugs, thus allowing them to drive tumor recurrence after therapy is suspended. Among the several mechanisms regulating cancer cell stemness, an important role seems to be played by the dysregulated expression of certain micro-RNAs (miRs). For instance, miR-21 is overexpressed in CRC and promotes tumor cell stemness, invasion and drug resistance via targeting several tumor suppressors, metastatic and apoptotic genes [36]. Other miRs have been linked to cancer stemness depending of the expression of certain tyrosine kinase receptors, such as EphB2 and EphA2, whose expression marks early and late CRC, respectively, EphB2 being a marker of staminality and EphA2 being expressed in the invasive CRC phase. A miRNAome-guided pathway analysis, in fact, defined two transcriptional signatures associated with EphB2 cells/early CRC phases and EphA2 cells/late CRC phases, with significant prognostic value. In particular, miR-31-5p and miR-31-3p overexpression were found in EphB2 highly-expressing cells whereas miR-432-5p was down-regulated in Epha2 highly-expressing cells. In addition, a gradual increase in miR-31-5p expression levels was observed with the progression of the TNM stage [37]. Finally, some miRNAs have been reported to impact cancer stemness and drug resistance via playing a pivotal role in the regulation of the epithelial-to-mesenchymal transition, which in turn is implicated in a wide array of malignant behaviors of cancers, including proliferation, invasion and metastasis [38]. Several studies have recently indicated that altered expression of certain miRs impacts on the response to antitumor agents also more directly. For example, miR-218 has a suppressing effect on BIRC5, which acts as an anti-apoptotic gene, and thymidylate synthase, which is a 5-FU target [39]. At variance, miR-34a expression is reduced in CRC and its re-expression attenuates the chemoresistance of colon cancer to 5-FU by inhibiting E2F3 and SIRT1. Notably, the miR-34a mimic MRX34 is the first synthetic miRNA entered into clinical trials (for an extensive review on the topic, see [36]). Other mechanisms of resistance to 5-FU, and chemotherapy in general, have been reported, such as altered expression of drug uptake carriers and efflux pumps, changes in phase I and phase II enzymes involved in drug metabolism (resulting in decreased pharmacological action either by an enhanced generation of inactive metabolites or diminished activation of prodrugs), altered expression levels of drug targets (such as thymidylate synthase, the target of 5-FU), enhanced capacity of tumor cells to repair the DNA damage usually induced by chemotherapeutic drugs or, to the contrary, dysfunction of the DNA damage sensing machinery and inability to undergo cell death after exposure to DNA damaging chemotherapy (such as upon TP53 loss) (for an extensive review on the topic, see [40]).

Besides a plethora of cancer cell inherent mechanisms, resistance to chemotherapy may be also elicited by the interaction of the tumoral cells with their microenvironment. For example, the hypoxic conditions characteristically found in the peripheral regions of the tumoral mass trigger the activation of the hypoxia-inducible factor-1α (HIF-1α) which induces the drug efflux pump MDR1. Accordingly, high expression of HIF1α and MDR1 detected by immunohistochemistry has been associated with a lower response to 5-FU in patients with advanced CRC [41]. Cancer-associated fibroblasts (CAFs) are essential components of CRC stroma that contribute to drug resistance by releasing cytokines, such as the previously mentioned TGFB. For example, in vitro and in vivo experiments using patient-derived cells showed that CAF-secreted TGFB acted synergistically with tumor cell-expressed HIF-1α to sustain 5-FU/oxaliplatin resistance via activation of the hedgehog pathway [42]. Additionally, different types of immune cells interact with cancer cells and other components of the tumor stroma through cytokine production, altering tumor growth and its response to drug therapy. Finally, due to the anatomical location of CRC, the gut microbiota has lately been demonstrated to contribute to the tumor microenvironment. In fact, several data clearly indicate that intestinal microbes not only impact on CRC initiation and progression by modulating intestinal inflammation, signaling pathways and local immune response, but also affect the response to chemotherapy and immunotherapy [43].

Since the beginning of the century, several targeted agents have been added to combinations with the above chemotherapies, in particular vascular endothelial growth factor (VEGF) inhibitors (such as the monoclonal antibodies bevacizumab and ramucirumab, the recombinant fusion protein aflibercept and the multi-kinase inhibitor regorafenib) and epidermal growth factor receptor (EGFR) inhibitors (such as the monoclonal antibodies cetuximab and panitumumab) [44]. Collectively, these targeted agents significantly improved median survival, but their use is still limited given that robust predictive biomarkers for anti-angiogenic treatment prioritization have not yet been identified. On the other hand, stratification criteria for selecting patients for anti-EGFR therapy have been identified but restrict the receiving patients only to those not possessing mutations in genes along the RAS/MAPK pathway (*KRAS*, *NRAS* and *BRAF*). Notably, mutant BRAF inhibitors are given to the subset of patients whose tumors express mtBRAF [44]. In addition, a combination using the BRAF inhibitors encorafenib and cetuximab may be used to treat patients with BRAF-mutated metastatic CRC who have received at least one previous treatment. Finally, immunotherapy with PD-1 targeting monoclonal antibodies can also be used in selected cases; pembrolizumab is used to treat unresectable or metastatic CRCs that are MSI-H or dMMR. In contrast, nivolumab can be administered to patients with MSI-H or dMMR metastatic CRC that has grown or spread after treatment with chemotherapy, either alone or in combination with ipilimumab (anti-CTLA-4 monoclonal antibody) (American Society of Clinical Oncology guideline, https://www.asco.org/; accessed on 10 April 2021).

The examples illustrated above are encouraging about the possibility to stratify patients in order to treat them according to a “precision medicine” approach. However, at the moment, only a restricted portion of CRC patients can benefit from this approach, given that the biomarkers identified so far to select patients, monitor the therapeutic response and/or predict the resistance to targeted drugs are still too few. Therefore, the need to identify additional biomarkers—detectable either in tissue or blood—to predict intrinsic or acquired resistance is very urgent, especially in the case of resistance to anti-EGFR drugs, given that they have been the first targeted drugs employed for the treatment of CRC and are so far the most used. In fact, encouragingly, new potential biomarkers are rapidly emerging from translational studies. Secondary *KRAS* mutations arise and are responsible for acquired resistance in approximately 50% of the patients who initially respond to cetuximab or panitumumab and in fact, mt-*KRAS* alleles can be detected in patients’ blood using highly sensitive circulating tumor DNA analysis methods before disease progression is clinically manifested [45,46]. Significantly, Bardelli et al. discovered that amplification of the *MET* proto-oncogene is responsible for de novo and acquired resistance to anti-EGFR therapy in a subset of wt-*RAS* CRCs. Notably, amplification of the *MET* locus was present in circulating tumor DNA before relapse was clinically evident. Finally, functional studies showed that MET activation confers resistance to anti-EGFR therapy both in vitro and in vivo [47]. Picardo et al. assessed the prognostic and therapy-response predictive values of the aberrant expression and methylation status of B4GALT1-a glycoprotein acting as a beta-1,4-galactosyltransferase in four cohorts of metastatic CRC cases. They reported that low expression level of B4GALT1 was significantly associated with poor cetuximab response, particularly in patients with wt-*RAS* tumors, thus suggesting it might be a novel biomarker for the prediction of cetuximab response, and as a specific and sensitive diagnostic circulating biomarker that can be detected in CRC [48]. Hasbal-Celikok et al. recently demonstrated that specific mutations in *AKT1* (E17K, E49K and L52R), as well as in *CTNNB1* (T41A, S45F and S33P), impaired the response to cetuximab in the presence of a wt-*RAS*. Moreover, these mutations were also associated with oxaliplatin, irinotecan, SN-38 and 5-FU resistance [49]. Another predictor of resistance to cetuximab in wt-*RAS* CRC has been indicated in EPH2A, a receptor involved in multiple cross-talks with other cellular networks, including EGFR, FAK, and VEGF pathways. In particular, in CRC, EPHA2 overexpression has been correlated with stem-like properties of cells, and its overexpression, together with overexpression of EGFR, was found to associate with poor response to cetuximab treatment. In addition, the same authors identified a molecular signature, comprising also EFNA1, PTPN12, ATF2 and mir-26b and mir-200, that was of prognostic significance in patients with stage I–III CRC and proposed it as a novel CRC prognostic biomarker [50]. Interestingly, a heavily dysregulated expression of several miRNAs has been found to be associated with drug resistance through various cellular and molecular mechanisms, related to apoptosis, cell cycle modification, alteration in drug targets, regulation of drug efflux transporters, epithelial-mesenchymal transition and cancer stem cells [51]. For example, high levels of miR-10/miR-125b, miR-345 and miR-199/miR-375 have been associated with cetuximab resistance, whereas overexpression of miR-302 restored the response to cetuximab (for an extensive review see Angerilli et al. [52]). miRNAs are attractive candidates as biomarkers to stratify patents since they are very stable molecules that can be easily detected in blood, urine and other bodily fluids given that they are not only present within cells, but are also actively secreted from cells, in RNA-binding multiprotein complexes and/or exosomes [53].

In conclusion, despite of the refinement of the classical chemotherapeutic approach and the targeted therapy approach, based on the identification of actionable targets and patient stratification criteria, resistance—both intrinsic and acquired—to drug treatment(s) remains one of the most significant challenges in the long-term management of incurable metastatic disease and eventually contributes to death as tumors accumulate means of evading treatment [54,55]. The identification of novel and more effective targets to be exploited alone or in combination with chemo-, targeted- or immunotherapy has therefore attracted a lot of efforts in the last two decades.

With the development of small interfering (si) and short-hairpin (sh)-RNA technologies, at the beginning of the century, and of the genome-wide CRISPR/Cas9 knockout screen, in the last decade, several screens have been performed, which has led to the identification of new actionable targets for overcoming drug resistance and/or being exploitable for synthetic lethality approaches in specific mutational settings. In addition, high-throughput chemical screenings led to the development of several small molecules effective in re-sensitizing drug-resistant tumor cells or acting as synthetic lethal agents for tumors with certain oncogenic mutations. Finally, given that the identification and validation of novel actionable targets and the development of new “targeted“ drugs is a highly costly and laborious process, several laboratories have also used a “drug re-positioning” or “re-purposing” approach, i.e., the finding of new indications for drugs in development or use in other diseases. This approach is relatively low cost and more swift since it makes use of already established preclinical and clinical knowledge.

This review aims to make an overview of the different approaches and to discuss relevant and promising targets identified so far for the treatment of CRC.

## 2. si/shRNA Screens

RNA interference is an evolutionary regulatory mechanism used by cells to control normal gene expression, where ~21–25-base-long specific double-strand small interfering (si) RNAs bind to their target mRNAs, triggering their degradation or hindering their translation into proteins. Experimentally, siRNAs can be synthesized as such or in the form of short hairpin (sh) precursors. Bioinformatic-assisted high-throughput production of large collections of siRNA targeting whole classes of proteins (kinases, phosphatases, Ub-ligase, etc.) allowed the use of si/shRNA libraries to perform large-scale loss-of-function studies as a powerful approach for therapeutic target identification in several fields, among them CRC.

### 2.1. Vascular Endothelial Growth Factor Receptor 1 (VEGFR1)

VEGFR1 is a tyrosine-protein kinase acting as a cell-surface receptor for VEGFA, VEGFB and PGF. It plays an essential role in developing embryonic vasculature, the regulation of angiogenesis, cell survival, cell migration, macrophage function, chemotaxis and cancer cell invasion [56]. VEGFR1 has a very high affinity for VEGFA and relatively low protein kinase activity; it may function as a negative regulator of VEGFA signaling by limiting the amount of free VEGFA and preventing its binding to VEGFR2 [57]. It has been reported that VEGFR-1 is present and functional on CRC cells, and activation by VEGF family ligands can activate processes involved in tumor progression and metastasis [58].

Naik and colleagues, by using a siRNA library targeting 691 known and predicted human kinases, uncovered an unanticipated non-endothelial role of VEGFR1 in the survival of cells addicted to WNT/beta-catenin signaling and demonstrated that VEGFR1 blockade is synthetic lethal in CRC cells with APC mutations [59]. Synthetic lethality occurs when a gene mutation (or treatment with a drug) non-lethal by itself results in the killing of the cell in the presence of another non-lethal gene mutation, such as a cancer-associated mutation. Therefore, targeting a synthetic lethal gene to a cancer-specific mutation should kill only cancer cells and spare normal cells without such a mutation [60]. A series of loss-of-function, genetic null and VEGFR inhibitor assays further confirmed that VEGFR1 is a positive regulator of WNT signaling that functions in a GSK3B-independent manner [61], making it an attractive target in those CRC tumors where APC function is lost.

Other evidence points to an important, non-angiogenic role of VEGFR1 in CRC; VEGFR1, together with VEGFR2 and their common ligand VEGF, is not expressed in normal human colonic cells, whereas its expression is high in CRC specimens. Furthermore, its stimulation by autocrine production of VEGF directly promotes colon cancer cell proliferation independently of the primary pro-angiogenic role [62]. Moreover, VEGF-stimulated VEGFR1 interacts with and stabilizes EGFR, leading to increased EGFR protein levels and prolonged expression on the cell membrane, whereas VEGFR1 blockade suppresses complex formation and decreases EGFR expression via a lysosome-dependent pathway, resulting in the suppression of proliferation activity [62]. As mentioned previously, for several years now the golden standard for anti-angiogenic therapy in the treatment of advanced CRC has been the monoclonal antibody bevacizumab that targets the VEGF–VEGFR pathway activated in tumor-associated neo-vasculature by sequestering all isoforms of VEGF-A, the most potent pro-angiogenic growth factor compared to other VEGFs [63]. Although the use of bevacizumab showed a good efficacy for CRC treatment, several studies reported various side effects of this anti-angiogenic molecule, the most frequent being hypertension—due to the blockade of VEGF-induced nitric oxide production in normal vessels—and proteinuria—likely due to inhibition of podocyte-derived VEGF [64]. Even though the primary intended target of bevacizumab is the tumor-associated neo-vasculature, it has been demonstrated that it can also directly act on CRC tumor cells; in fact, it has been shown that bevacizumab can induce metastatic behaviors in in vitro and mice models of CRC [65]. The major signal transducer upon VEGF-A binding is VEGFR2, whose expression and activity on endothelial cells are 10 times more than those of VEGFR1 [63], thus implying that bevacizumab mainly inhibits VEGFR2-mediated signaling in angiogenic cells. However, VEGFR2, in its phosphorylated form, is also largely expressed in colon cancer cells (but not in normal colonic cells) where its levels are significantly associated with a tumor diameter > 6 cm (*p* = 0.04) and poor histological differentiation (*p* = 0.004) [57]. Therefore, VEGFR2 is not a vasculature-restricted receptor but has an additional role in cancer cell biology itself, likely via an autocrine VEGF/VEGFR2 loop leading to cell proliferation, migration and resistance to apoptotic stimuli [66].

Based on what was discussed above, the exploitation of VEGFR1 as a target in CRC cells with APC mutations should lead to a better therapeutic window than VEGF-A targeting, and specific inhibition of the tumor-expressed VEGFR1 and its autocrine loops may be a yet to be explored therapeutic option.

### 2.2. GSK3B

GSK3B is an evolutionarily conserved serine/threonine kinase, functioning in numerous cellular processes, including cell proliferation, DNA repair, cell cycle, signaling and metabolic pathways. GSK3B is implicated in different diseases including inflammation, neurodegenerative disease, diabetes and cancers [67,68].

Given that resistance to drug treatment(s) remains the main reason of the therapeutic failure ultimately responsible for the death of CRC patients, our lab aimed to identify specific targets for the treatment of 5-FU-resistant tumors by using a kinase-directed sh-RNA library and HCT116p53KO CRC cells as a model for drug-resistance. A p53-null background was used as a model, given that p53 activity is either lost or compromised in most tumors, which abolishes the apoptotic response to many anticancer agents. By this approach several kinases were identified whose silencing bypassed 5-FU resistance due to loss of p53, among which was GSK3B [69]. Downregulation of GSK3B in various 5-FU-resistant p53-null CRC cell lines was shown to abolish cell viability and colony growth after drug treatment without affecting the cell proliferation or cell cycle of untreated cells. Because p53 function is compromised in the vast majority of human cancers and caspase-dependent apoptosis is frequently impaired in tumors, a very interesting finding was that upon GSK3B inhibition, 5-FU bypassed the need of p53 to induce cell death, and tumor cells died by caspase-independent necroptosis. In vivo studies using 5-FU-resistant xenografts confirmed that targeting GSK3B re-sensitized tumors to 5-FU. Finally, tissue microarray analysis of CRC samples from a cohort of 5-FU-treated patients revealed that GSK3B is significantly more activated in drug-resistant versus responsive patients. On the whole, these data led us to conclude that GSK3B inhibition in combination with chemotherapy may represent a molecularly targeted approach to treat resistant CRCs.

Thorne et al. performed a GSK3 modifier screen across the known human kinome by transfecting a kinase-targeted siRNA library in colon cells, followed by treatment with a GSK3 inhibitor. They identified several kinases whose loss-of-function combined with GSK3 inhibition impacted on cell viability, among which were BRAF, VEGFR2 and PLK1 [70]. Notably, BRAF and VEGFR2 inhibitors are already used in clinics to treat advanced CRCs, and PLK1 inhibitors are in clinical trial, suggesting that combining GSK3 targeting with more and diverse targeted treatments would be an innovative therapeutic approach to be explored.

Recently, Park and colleagues reported that GSK3B might be an actionable target in a subset of CRCs harboring a PIK3CA mutation (15 to 20% of all CRCs) and for this reason, be resistant to dual PI3K/mTOR inhibition (by the specific inhibitor gedatolisib). They, in fact, demonstrated that gedatolisib-resistant cell lines expressed high levels of active GSK3B and harbored the same frameshift mutation (c.465_466insC; H155fs*) in TCF7, which encodes a positive transcriptional regulator of the WNT/beta-catenin signaling pathway. Inhibition of GSK3B effectively reduced signaling downstream of mTOR and through the WNT/beta-catenin pathway. Notably, GSK3B inhibition rendered the resistant cell lines sensitive to gedatolisib, both in vitro and in mouse xenografts, suggesting that GSK3B targeting may be a strategy to overcome the resistance of PIK3CA- and TCF7-mutant CRC to PI3K/mTOR-targeted therapies [71]. It is important to note that mTOR exists in two complexes, mTORC1 and mTORC2, which can be both activated by PI3K. However, only mTORC1 can be activated directly by AKT, whereas mTORC2 can be a relay between PI3K and AKT so that AKT activation can be both upstream and downstream of mTOR, depending on the complex (Figure 4). In addition, mTORC2 can also be activated independently of PI3K (for example, via AMPK) and its activation can be limited by a negative feedback loop acted by mTORC1, thereby rendering the inhibition of the PI2K/AKT/mTOR axis particularly complex and prone to unwanted side effects.

Finally, a more general role for GSK3B in drug resistance can be proposed given that several studies indicated GSK3B as an actionable target for the treatment of drug-resistant carcinomas derived not only from colon but also from kidney [72], pancreas [73,74,75], endometrium [76] and for glioblastomas [77,78,79]. Therefore, it seems that GSK3B participates in multiple molecular pathways used by various cancer types to evade chemotherapy, radiotherapy and targeted therapies [80]. Interestingly, 9-ING-41, a novel GSK3B inhibitor developed by Actuate Therapeutics, is currently undergoing phase 1 and phase 2 trials as a single agent and in combination with cytotoxic agents, in patients with refractory cancers, including CRCs.

### 2.3. p65kDa Isoform of the Bruton Tyrosine Kinase (p65BTK)

BTK is essential for B-cell proliferation/differentiation, and for a long time it was generally believed that its expression and function were limited to bone marrow-derived cells, either normal or tumoral. In fact, specific inhibitors such as ibrutinib and acalabrutinib are already therapeutically used for certain B-cell malignancies, where BTK is overexpressed/hyperactivated, and several other specific BTK inhibitors are currently in clinical trials for B-cell malignancies and auto-immune diseases characterized by abnormal B-cell proliferation [81,82].

From the same kinase-directed shRNA screen described in the previous paragraph, our laboratory identified a novel isoform of the kinase, which we dubbed p65BTK from its apparent molecular weight [83]. We found that p65BTK is abundantly expressed in colon carcinoma cell lines and tumor tissue samples, where its expression correlates with ERK1/2 activation and its inhibition affects the growth and survival of colon cancer cells in vitro. Compared to the already known BTK isoform expressed in bone marrow-derived cells (molecular weight: 77 kDa), p65BTK mRNA is transcribed from a different promoter, contains a different first exon and its translation produces a protein lacking the first 86 *N*-terminal amino acids. Remarkably, structural studies indicated that the lack of the *N*-terminal leads to increased levels of spontaneous p65BTK activation [84]. Despite the very low expression of its mRNA, the abundance of the protein is tightly controlled at the translational level through heterogeneous nuclear ribonucleoprotein K (hnRNPK)-dependent and internal ribosome entry site (IRES)-driven translation, and occurs downstream of the MAPK pathway. Moreover, we found that p65BTK is endowed with strong transforming activity that depends on active ERK1/2, and its inhibition abolishes RAS transforming activity. Therefore, we demonstrated that BTK, via p65BTK expression, is a novel and powerful oncogene acting downstream of the RAS/MAPK pathway and is likely a promising therapeutic target [83]. We then demonstrated that p65BTK silencing or chemical inhibition overcame 5-FU resistance of CRC cell lines and patient-derived organoids and significantly reduced the growth of xenografted tumors. Mechanistically, we showed that blocking p65BTK in drug-resistant cells abolished a 5-FU-elicited TGFB1 protective response and triggered E2F-dependent apoptosis, thus giving a proof-of-concept for the use of BTK inhibitors in combination with 5-FU as a novel therapeutic approach in CRC patients [85]. Clinically, we confirmed p65BTK being a strong candidate target by quantifying its expression in three different cohorts of CRC patients for a total of 254 patients. We found that p65BTK expression levels significantly increased with histological tumor grade, suggesting an inverse correlation between p65BTK expression levels and cellular differentiation, and confirmed that > 70% of CRC samples showed medium-to-strong intensity (++/+++) of p65BTK staining [32]. Finally, we performed univariate analysis on a retrospective study which evaluated 87 consecutive stage III CRC patients treated at the National Cancer Institute of Aviano (1999–2017), and determined that patients highly expressing p65BTK (IHC intensity 3 and ≥ 80%) had the worst prognosis in terms of DFS (HR: 6.23; *p* = 0.005; 95% C.I. 1.75–22.79) and OS (HR: 2.54; *p* = 0.025; 95% C.I. 1.12–5.76) [85].

Notably, we found p65BTK expression also in glioblastoma [86] and lung tumors, where we demonstrated that p65BTK is a novel potential actionable target in mt-*KRAS*/wt-*EGFR* in non-small cell lung carcinomas [87]. Finally, unpublished data from our laboratory indicate p65BTK as an actionable target also in ovarian cancer (Conconi et al., manuscript in preparation) and in melanomas (Bonomo et al., submitted) suggesting that p65BTK is likely to be a very promising therapeutic target not only in CRC but also in several solid tumors.

### 2.4. Protein Kinase C Delta Type (PRKCD)

PRKCD is a calcium-independent, phospholipid- and diacylglycerol-dependent serine/threonine protein kinase that plays contrasting roles in cell death and cell survival by functioning as a pro-apoptotic protein during DNA damage-induced apoptosis but acting as an anti-apoptotic protein during cytokine receptor-initiated cell death. It is involved in tumor suppression as well as the survival of several cancers [88,89].

Sun and colleagues took advantage of RNAi technology to perform a focused screening. They used a custom siRNA library targeted against a subset of 151 highly mutated candidate genes (informally referred to as driver alterations or candidate cancer genes, CAN-genes), identified by sequencing the CRC genome [90] as potential driver genetic alterations directly involved in CRC tumorigenesis. By this approach they focused on the CAN-genes to identify those alterations that cause oncogene or non-oncogene addiction in CRC and they identified PRKDC as an essential gene for CRC cell growth/survival both in vitro and in vivo [91]. They demonstrated that transient knockdown of PRKDC reduced cell proliferation/survival in CRC cell lines and induced apoptosis partially through inhibiting AKT activation. Moreover, PRKCD silencing sensitized CRC cells to chemotherapeutic agents interfering with DNA replication, such as 5-FU and oxaliplatin, both of which are commonly used for CRC chemotherapy. In addition, inducible knockdown of PRKDC inhibited tumor growth in vivo. Finally, PRKDC was up-regulated in cancerous tissues compared with normal tissues and patients with high PRKDC expression showed poorer overall survival [91].

The importance of PRKDC in CRC biology is further underscored by findings from Dietlein et al. who, using a completely different experimental approach, uncovered a druggable synthetic lethal interaction between MSH3 and PRKDC [92]. In this study, a large-scale cell line-based approach was employed to identify cancer cell-specific mutations that are associated with PRKDC dependence. To this end, the authors profiled the mutational landscape across 1319 CAN-genes of 67 distinct cell lines and identified numerous genes involved in homologous recombination-mediated DNA repair (including BRCA1, BRCA2, ATM, PAXIP and RAD50), among which the MMR gene MSH3, mutated in ∼40% CRCs, emerged as the most significant predictor of PRKDC addiction. Accordingly, PRKDC inhibition robustly induced apoptosis in MSH3-mutant cell lines in vitro and displayed remarkable single-agent efficacy against MSH3-mutant tumors in vivo. However, loss of MSH3 is somewhat secondary, due to MMR-deficiency, and frequently detectable in MLH1-deficient tumors. Therefore, Hinrichsen and colleagues examined the expression of MLH1, MSH2, MSH6 and MSH3 in different MMR-deficient and proficient cell lines and determined their sensitivity to PRKDC inhibition, and found that that MLH1 and/or MSH3-deficient cells exhibited a significantly higher sensitivity to PRKDC inhibition than MMR-proficient cells, and that overexpression of MLH1 in MLH1-deficient cells resulted in a decrease in cell sensitivity. Since the molecular testing of colon tumors for MLH1, and not MSH3 expression, is a clinical standard, they proposed that MLH1 is a much better marker to be assessed for a more significant number of patients to benefit from PRKDC inhibition [93].

Targeting PRKDC might be beneficial for CRC therapy, not only in a context of the synthetic lethality approach, but also to overcome resistance to chemotherapy. Irinotecan specifically targets topoisomerase I and is used to treat CRC, but only 13–32% of patients respond to the therapy, a rapid rate of topoisomerase I degradation in response to irinotecan being the cause of resistance. Ando et al. demonstrated that the deregulated PRKDC cascade ensures rapid degradation of topoisomerase I and that this is at the core of the drug resistance mechanism to topoisomerase I inhibition [94]. In particular, they found that PRKDC phosphorylates topoisomerase I on serine 10, which is subsequently ubiquitinated by BRCA1 and marked for proteasomal-dependent degradation. A higher basal level of phospho-topoisomerase I ensures its rapid degradation and, consequently, resistance to irinotecan treatment. Importantly, PTEN negatively regulates PRKDC kinase activity in this pathway and PTEN deletion provides a PRKDC-dependent higher phospho-topoisomerase I degradation. Notably, in CRC, PTEN inactivation may occur via multiple mechanisms such as genomic mutations (2%), loss of protein expression (34.3%), promoter hypermethylation (27.3%) and decreased DNA copy number (18.2%). Moreover, the frequency of loss of PTEN expression increases from 20% in stage I to 56.9% in stage IV disease [95]. Thus, PRKDC inhibition in addition to irinotecan treatment might be particularly beneficial in colon cancers with PTEN inactivation.

On the whole, these data suggest that the addition of PRKDC inhibitors to classic chemotherapy might be beneficial for a high percentage of CRC patients, mainly because potent PRKDC inhibitors (AZD7648, nedisertib) are currently entering early clinical trials.

### 2.5. Targets Exploitable in KRAS-Mutated Colon Cancers

The therapeutic significance of KRAS mutation in CRC is well defined given that this renders these tumors resistant to anti-EGFR therapies [96,97,98,99]. Despite this prevalence and its prominent status as a cancer drug target, molecules aimed at disrupting KRAS signaling have proven challenging, and mutant KRAS protein has remained an intractable therapeutic target for over two decades. Only very recently, the first small molecule that binds one form of mutant KRAS with high specificity and sensitivity, inhibiting the protein, has been described [100]. Given KRAS’s “undruggability”, with the advent of RNA interference technology several studies have been carried out to identify synthetic lethal genetic interactions in the context of CRC-bearing mutant KRAS.

#### 2.5.1. Polo-Like Kinase-1 (PLK1)

PLK1 is a conserved serine/threonine protein kinase that performs several critical functions as a regulatory protein throughout the M phase of the cell cycle, being involved in spindle assembly and mitosis. Moreover, it plays an important role in maintaining genome stability and the DNA damage response [101].

Luo et al. undertook a genome-wide RNAi screen to identify synthetic lethal interactions with the KRAS oncogene using an enrichment approach [102]. First, they screened the parental KRAS^WT/G13D^ DLD-1 cells (where only one allele carries the mutation) and the isogenic *KRAS*^WT/-^ DLD-1 control cells with a library of 74,905 retroviral shRNAs targeting 32,293 unique human transcripts. The relative abundance of each shRNA over time was analyzed by microarray hybridization to identify those that were antiproliferative and thus depleted from the population. A lethality signature comprising a subset of 379 RAS synthetic lethal (RSL) shRNAs, targeting 368 genes, was then established, identifying those shRNAs showing selective depletion in the mut- but not wt-*KRAS* cells. A total of 320 candidate RSL shRNAs from the primary screen were used for a secondary screen, eventually leading to the identification of 83 shRNA (26%), targeting 77 genes, preferentially decreasing the fitness of mut- vs. wt-*KRAS* cells. To rule out cell-line-specific effects, the secondary screen was repeated in a second isogenic pair of colorectal cancer cell lines, HCT116 KRAS^WT/G13D^ and HCT116 *KRAS*^WT/-^; 50 of 68 tested shRNA (73.5%) also showed synthetic lethality in the HCT116 cells, indicating that the majority of candidate RSL shRNAs were likely to interact genetically with KRAS. Pathway analysis revealed that the targeted genes were involved in several different biological processes such as ribosomal biogenesis and translation control, protein neddylation and sumoylation pathways, RNA splicing and regulation of mitosis. The authors then focused on PLK1, since it plays a key role in mitosis, and its activity is often deregulated in cancer cells and inhibitors against PLK1 have been developed as potential cancer therapeutics. They determined that mut-*KRAS* cells are hypersensitive to mitotic stress and to PLK1 inhibition both in vitro and in vivo. Notably, clinical trials with the PLK1 inhibitor onvansertib [103] given together with bevacizumab and FOLFIRI are currently ongoing as a second-line treatment of metastatic CRC in patients with mut-*KRAS.*

The exploitability of PLK1 as a therapeutic target in KRAS CRCs has been corroborated by other experimental evidences independently gathered by other groups and using different approaches. Denkert’s group reported that whereas normal colon mucosa and adenomas showed only a weak expression of PLK1, 66.7% of carcinomas showed instead a strong expression of PLK1. Notably, overexpression of PLK1 correlated positively with Dukes stage, tumor stage and nodal status. Additionally, PLK1 expression was a prognostic marker; in fact, patients with PLK1-positive tumors showed a rate of 65% survival after 5 years compared to 86% survival in the PLK1-negative group. In a subgroup without distant metastasis, the 5-year survival was reduced from 89% in the PLK1-negative group to 70% in the PLK1-positive group [104].

Tumor-initiating cells are responsible for tumor maintenance and relapse in solid and hematologic cancers. In colon cancer, this tumorigenic population can be found in a rapidly proliferating state in vitro and in vivo, both in human tumors and mice [105]. Interestingly, PLK1 inhibitors demonstrated maximal efficiency over other targeted compounds and chemotherapeutic agents in inducing the death of colon cancer-initiating cells in vitro. In vivo, PLK1 inhibitors killed CD133(+) colon cancer cells, leading to complete growth arrest of colon cancer stem cell-derived xenografts, whereas chemotherapeutic agents only slowed tumor progression. While chemotherapy treatment increased CD133(+) cell proliferation, treatment with PLK1 inhibitors eliminated all proliferating tumor-initiating cells. Quiescent CD133(+) cells that survived the treatment with PLK1 inhibitors could be killed by subsequent PLK1 inhibition when they exited from quiescence [105]. These results further reinforce the importance of targeting PLK1 in the treatment of CRC, especially in the case of drug-resistant tumors.

Besides combination with classical chemotherapy and targeted therapy (such as the previously cited GSK3B inhibitor) [70], PLK1 inhibitors proved to be helpful when administered 24 h before irradiation (but not after) because they caused cells to accumulate in G2/M and resulted in increased radiosensitivity [106].

Finally, PLK1 seems to be an exploitable target not only in mut-*KRAS* CRCs but also in the context of p21 loss [107], an event occurring in 79% of the patients, where it is associated with more prolonged survival among patients ≥60 years old, whereas it is associated with shorter survival among patients <60 years old [108].

#### 2.5.2. The Proteasome

The eukaryotic 26S proteasome is a large multisubunit complex that, under normal conditions, degrades most proteins in the cell. The 26S proteasome can be divided into two subcomplexes: the 19S regulatory particle and the 20S core particle. Most substrates are first covalently modified by ubiquitin, which then directs them to the proteasome. The function of the regulatory particle is to recognize, unfold, deubiquitylate and translocate substrates into the core particle, which contains the proteolytic sites of the proteasome [109]. The proteasome degrades most cellular proteins in a controlled and tightly regulated manner, thereby controlling many processes, including cell cycle, transcription, signaling, trafficking and protein quality control. Proteasomal degradation is vital in all cells and organisms, and dysfunction or failure of proteasomal degradation is associated with diverse human diseases, including cancer and neurodegeneration [110].

Steckel and colleagues undertook a large-scale siRNA screen in the mut-*KRAS* HCT116 cells vs. HKE-3 wt-*KRAS* cells, thus performing a classic synthetic lethality screen. Calculation of an apoptosis ratio between the two cell lines allowed them to identify targets whose silencing lead to much stronger induction of apoptosis in HCT116 than HKE-3. A secondary screen using the top 52 hits from the primary screen was performed to evaluat the ability to kill a panel of 28 tumor cell lines from various cancer types, comprising 14 that carried an activating KRAS mutation and 14 that did not (Table 1). Cell lines were selected from the NCI60 tumor cell line collection among those most frequently harboring KRAS mutations: 11 CRC cell lines (six harboring mut-*KRAS*), 9 lung cancer cell lines (four harboring mut-*KRAS*), 2 mut- and 1 wt-*KRAS* pancreatic cancers, 1 mut- and 2 wt-*KRAS* ovarian cancers and 1 mut- and 1 wt-*KRAS* stomach cancers [111].

The screen identified a strikingly high number of proteasome components; indeed, from a total of 13 proteasome components present in the screen, eight ranked within the top 300 genes and four in the final 52 selected genes. In fact, proteasomal protein PSMD14 showed significant selectivity for mut-*KRAS* cell lines in the apoptosis assay with the large panel of 28 cell lines, and chemical inhibition of proteasome function using the licensed cancer drug bortezomib (in the clinic for the treatment of multiple myeloma) confirmed a selective loss of cell viability, associated with the induction of apoptosis, in mut-*KRAS* cells. In contrast to these findings from the screening, a previous clinical trial showed that single agent bortezomib is inactive in metastatic colorectal cancer [112], whereas a phase I study of bortezomib in combination with a FOLFOX regimen in patients with advanced colorectal cancer reported that amongst 13 evaluable patients, five had a partial response (38.5%), five had a stable disease (38.5%) and three patients progressed (23%) [113]. However, in neither case were patients genotyped for *KRAS*, leaving open the possibility that a synthetic lethal effect of proteasome inhibition might be clinically relevant. Much of the literature shows that, in vitro, cell lines derived from solid tumor cells are sensitive to bortezomib as multiple myeloma cells. However, since pharmacodynamics data obtained in clinical trials show that bortezomib equally inhibits proteasomes in solid tumors and blood, a possible explanation for the lack of response to proteasome inhibitors in solid tumors in vivo is insufficient potency. Finally, the most recent findings indicate that inhibitors targeting different active centers of the proteasome and diverse types of proteasomes may achieve different therapeutic benefits and have different potency [114]. For example, specific inhibition of the immunoproteasome subunit LMP7 (one of the subunits whose synthesis is specifically stimulated by pro-inflammatory cytokines, such as IFN-γ or TNF, leading to the formation of the immunoproteasome) has been shown to interfere with CRC development; in fact, treatment with the LMP7 inhibitor ONX 0914 blocked tumor initiation and progression in either chemically-induced (AOM/DSS) or transgenic mouse models (ApcMin/+) of colon carcinogenesis [115].

All these data suggest that indeed the proteasome(s) appears to be an attractive therapeutic target in CRC, and more studies are needed using different proteasome inhibitors in molecularly defined settings. For instance, the use of mut-*KRAS* patient-derived organoids and xenografts would represent an invaluable preclinical model to test novel proteasome inhibitors already in clinical trials for multiple myeloma, such as ONX 0914, carfilzomib, ixazomib and oprozomib. Another option to test would be the combination of proteasome inhibitors with DNA-damaging drugs as indicated by the findings that the pre-treatment with gemcitabine, irinotecan and doxorubicin sensitized mut-*KRAS* CRC cells to a subsequent exposure to low concentrations of proteasome inhibitors [111].

#### 2.5.3. Ubiquitin-Specific Protease 39 (USP39)

Deubiquitinases (DUBs), a large group of proteases with the ability to hydrolyze the peptide and isopeptide bonds that link ubiquitin chains to target proteins, can be subdivided in six families, cysteine proteases USP being the largest group of DUBs. In particular, USP39 is a pseudo-protease devoid of deubiquitinase activity [116] but its function is essential in pre-mRNA splicing as a component of the U4/U6-U5 tri-snRNP, one of the building blocks of the precatalytic spliceosome [117]. Moreover, it regulates AURKB mRNA levels, and thereby plays a role in cytokinesis and in the spindle checkpoint [118].

Given that the number of human malignancies in which DUBs show changes in their expression levels or are mutated has substantially grown over the last few years, Fraile and colleagues, using KRAS-dependent cancer cells as a model, performed an shRNA-based synthetic lethal screen using a custom library of shRNAs targeting most DUBs. Initially, the screen was performed and validated on lung cancer cells whose viability depended on KRAS and subsequently, the top-scoring hit, i.e., USP39, was further validated in CRC cells, given that many of them are also dependent on mut-*KRAS* signaling. As models, two pairs of isogenic cell lines (derived from DLD-1 and HCT116) differing only in presence or absence of a KRAS mutation were used; in both models USP39 downregulation selectively decreased the growth of mut-*KRAS* cells, both in vitro and in vivo [119]. To further explore the association between KRAS and USP39 dependence in various tumor types, the authors also examined data derived from a genome-wide shRNA screen in 216 cancer cell lines from multiple tumor types (Project Achilles) [120], highlighting a significant positive correlation between the antiproliferative effects of silencing both genes. Moreover, USP39 silencing affected the pre-mRNA splicing efficiency of several genes directly associated with KRAS-related processes and selected by gene set enrichment analysis (GSEA), performed after RNA-seq of total RNA from KRAS-dependent vs. KRAS-independent HCT116 cell lines, transduced with control or USP39-specific shRNAs. Accordingly, mut-*KRAS* were much more sensitive than wt-*KRAS* cells to the treatment with splicing inhibitors sudemycin D1 and D6 and FR901464. Finally, using publicly available datasets, significant up-regulation of both KRAS and USP39 was found in lung cancer patients where high expression levels of USP39 were also associated with short survival.

The importance of USP39 in cancer cell proliferation, survival, migration and resistance to chemotherapy has been reported in a wide variety of tumors beside CRC [121,122] and lung cancer [123], such as hepatocellular carcinoma [124,125,126], ovary carcinoma [127,128], osteosarcoma [129,130], glioma [131], melanoma [132], gastric [133] and pancreatic cancer [134] and renal cell carcinoma [135]. To date no clinically relevant USP39 inhibitors are available; however, several candidates are under study at the preclinical level and drugs specifically targeting other components of the spliceosome machinery are also being investigated preclinically [136,137]. It, therefore, might be interesting to test them in the patient-derived tumor models mentioned in the previous paragraph, either alone or in combination with chemo- or targeted therapy.

### 2.6. Targets Exploitable in BRAF-Mutated Colon Cancers

*BRAF* status is believed to be responsible for the 12–15% of patients who fail anti-EGFR [138,139]. Trials investigating the effect of drugs specifically targeting BRAF^V600E^ mutants, such as vemurafenib, given as monotherapy, have failed in mut-*BRAF* colorectal cancers [140], even though they proved to be effective in melanomas harboring the same mutation [141]. In particular, it has been shown that the unresponsiveness of colon cancer to BRAF^V600E^ inhibition occurs through feedback activation of EGFR [142,143]. The more recent trials with BRAF/EGFR double-therapy or BRAF/MEK/EGFR triple-therapy have shown some increased response rates but at the cost of increased toxicity, and patients ultimately develop resistance, due to MAPK pathway reactivating alterations [144]. Therefore, as in the case of *KRAS*, identifying vulnerability to exploit for therapy, synthetic lethal screens have been performed in the context of CRC models bearing mut-*BRAF*.

#### 2.6.1. Protein Tyrosine Phosphatase Non-Receptor Type 11 (PTPN11)

PTPN11, also known as SHP2, represents a common node downstream of receptor tyrosine kinases (RTK) and is required for RAS activation, although the mechanisms by which SHP2 contributes to RAS activation have not been completely elucidated. Upon RTK engagement SHP2 acts upstream of the RAS-GEF SOS, which promotes RAS exchange of GDP for GTP, thus leading to RAS activation and the engagement of the RAF/MEK/ERK pathway [144].

To search for phosphatases whose knockdown induces sensitivity to BRAF inhibition, Prahallad and colleagues performed, in BRAF-mutant CRC cells treated with vemurafenib, an shRNA-screen targeting 298 phosphatases or phosphatase-related genes; PTPN11 was identified as a central node in intrinsic and acquired resistance to BRAF^V600E^ inhibition [145]. In fact, suppression of PTPN11 in vemurafenib-resistant CRC cells prevented feedback activation of EGFR/MEK/ERK signaling usually occurring as a mechanism of resistance to BRAF inhibition [145]. It is important to note that PTPN11 knockout by itself did not affect cell proliferation in the absence of vemurafenib, consistent with the notion that PTPN11 is upstream of mut-*BRAF*. In contrast, when PTPN11 knockout cells (obtained by using an inducible CRISPR-Cas9 vector) were treated with vemurafenib, massive apoptosis occurred by 48–72 h and the same effect was observed when treating parental cells with a combination of a specific PTPN11 inhibitor with vemurafenib. The synthetic lethal effect of PTPN11 loss with BRAF inhibition was then confirmed in vivo, in a xenograft model where tumor growth was almost completely suppressed [145]. Moreover, PTPN11 loss suppressed colony growth of cell lines harboring specific activations in different RTKs, such as *EGFR* amplification, *EGFR* mutation and an *EML4–ALK* translocation, clearly indicating that RTK engagement needs PTPN11 for signal transduction and MAPK pathway activation. Finally, analyzing biopsies from BRAF^V600E^ mutant melanoma patients (*n* = 4) who had progressed upon vemurafenib treatment, these authors found that PTPN11 phosphorylation at Y542 can serve as a biomarker to identify tumors with RTK-driven acquired resistance to BRAF inhibitors.

Notably, PTPN11 serves as a central hub to connect several intracellular oncogenic signaling pathways other than the RAS/RAF/MAPK, such as JAK/STAT, PI3K/AKT and PD-1/PD-L1 pathways [145]. Therefore, the development of specific inhibitors for its targeting has been pursued for more than a decade, and four SHP2 allosteric inhibitors have recently entered clinical trials for the treatment of solid tumors.

#### 2.6.2. The Unfolded Protein Response (UPR)

Proteins requiring post-translational modifications are processed in the endoplasmic reticulum where an accumulation of incorrectly folded proteins can trigger the unfolded protein response (UPR). In mammalian cells, UPR performs three functions: adaptation, alarm and apoptosis. During adaptation, the UPR tries to re-establish folding homeostasis by inducing the expression of chaperones that enhance protein folding. Simultaneously, global translation is attenuated to reduce the ER folding load, while the degradation rate of unfolded proteins is increased [146]. If these steps fail, the UPR induces a cellular alarm and the mitochondrial-mediated apoptosis program. UPR malfunctions have been associated with a wide range of disease states, including tumor progression and diabetes, as well as immune and inflammatory disorders [146]. In particular, glucose-regulated protein 78 (GRP78) is a chaperone heat-shock protein that is the master of the UPR; its primary function is to bind to the unfolded proteins to prevent misfolding and when the load of the unfolded protein is too high, it drives the cell to autophagy or apoptosis [147].

A combined approach to identify novel actionable targets in the mut-*BRAF* CRCs was used by Forsythe and colleagues [148]. They first performed differential gene expression and pathway analyses of untreated stage II and stage III mut-*BRAF* CRCs (discovery set: *n* = 31; validation set: *n* = 26) and identified five top pathways significantly upregulated in the poor prognostic mut-*BRAF* CRCs: cholesterol biosynthesis pathway, the geranylgeranyldiphosphate biosynthesis and mevalonate pathway, G-protein-coupled receptor signaling and the UPR. They then transfected a siRNA library targeted to the differentially expressed genes in four diverse mut-*BRAF* CRC cell lines, finding that only the targeting of HSPA5—the gene encoding the master regulator of UPR GRP78—had a significant inhibitory effect on the survival of cell lines. They then confirmed the involvement of GRP78 and the UPR in regulating the survival of mut-*BRAF* CRC cells by using HA15, a small molecule inhibitor against GRP78 that has been previously reported to display anti-cancerous activity on melanoma cells, including cells isolated from patients and cells that developed resistance to BRAF inhibitors, and other liquid and solid tumors. Moreover, HA15 also exhibited strong efficacy in xenograft mouse models with melanoma cells either sensitive or resistant to BRAF inhibitors [148]. Forsythe and colleagues also demonstrated that oncogenic BRAF and activated MEK1/2-ERK1/2 signaling results in enhanced protein synthesis and chronic ER stress, rendering mut-*BRAF* CRC cells susceptible to apoptosis following treatment with acute ER stress activators such as HA15. Finally, to demonstrate that mut-*BRAF* CRC cells are especially sensitive to UPR, they were treated with two inhibitors of protein degradation pathways, the proteasomal inhibitor carfilzomib (CFZ) and the aggresome inhibitor ACY-1215, both of which are in clinical development and have been found to result in an overload of misfolded/damaged proteins and ER stress. Both drugs affected the viability of mut-*BRAF*, but not wt-*BRAF*, CRC cells, and when used in combination they triggered massive apoptosis in in vitro models and significant tumor reduction in xenograft models [149]. Notably, two molecules that have been shown to preferentially decrease the expression of GRP78 in tumor cells and ER-stressed cells when compared to normal cells, BOLD-100 and NKP-1339, are being tested in phase I clinical trials in CRC patients.

#### 2.6.3. Splicing Factor Proline and Glutamine-Rich Protein (SFPQ)

SFPQ is a multifunctional protein playing several roles in cell biology. It is an essential pre-mRNA splicing factor required early in spliceosome formation and for splicing catalytic step II, and is involved in the regulation of signal-induced alternative splicing. Moreover, it binds to DNA by forming an SFPQ-NONO heterodimer, which participates in homologous DNA pairing and in DNA non-homologous end joining (NHEJ) required for double-strand break repair. Finally, SFPQ is involved in transcriptional regulation as a transcriptional activator [150,151]. Since monotherapies blocking downstream components of the MAPK signaling pathway have been unsatisfactory in CRC because of pathway reactivation, Klotz-Noack and colleagues hypothesized that interference with nuclear proteins activated by MAPK might open a window of opportunity for precisely eliminating MAPK-driven CRCs. To test this option, they performed an shRNA screen to probe MAPK targets encoding nuclear and/or DNA-binding factors in isogenic CRC cell lines inducible for oncogenic BRAF. By this approach, they identified SFPQ as a novel factor synthetically lethal with BRAF^V600E^. In fact, knockdown of SFPQ and the expression of BRAF^V600E^ strongly decreased colony numbers and sizes in in vitro systems and led to dramatic shrinkage of tumors in xenograft models [152]. In particular, they showed that SFPQ depletion decreases proliferation and specifically induces S-phase arrest and apoptosis, not only in BRAF^V600E^-driven CRC cells, but also in melanoma cells. Mechanistically, they demonstrated that SFPQ loss in mut-*BRAF* cancer cells triggers the CHK1-dependent replication checkpoint, results in decreased numbers and reduced activities of replication factories and increases collision between replication and transcription. Accordingly, BRAF^V600E^-mutant cancer cells and organoids were shown to be sensitive to combinations of CHK1 inhibitors and chemically induced replication stress by means of low doses of hydroxyurea, suggesting the use of HU/CHK1 inhibition as a treatment option for BRAF^V600E^-mutant multi-therapy-resistant CRCs.

**Figure 5:**
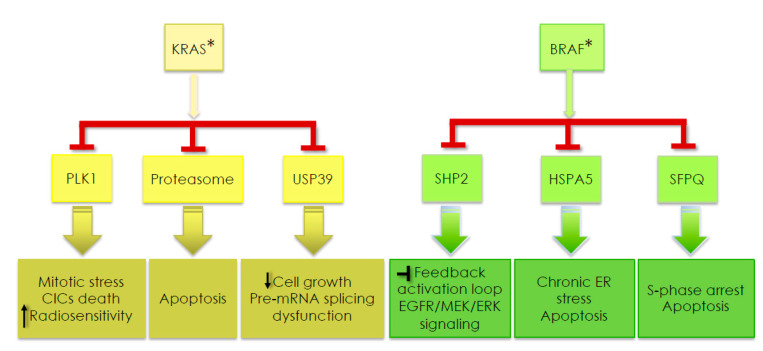
Genes identified by siRNA/shRNA screen as synthetic lethal in *KRAS-* and *BRAF*-mutated CRCs. CIC: cancer-initating cells. *: mutated. Arrows indicates downstream events. Red lines indicate the blockade of the gene product either by si/RNA or by chemical inhibitors.

A summary of genes synthetic lethals with mutated *KRAS* and *BRAF* and the downstream events occurring after their blockade is given in Figure 5

Even though not identified by a large-scale loss-of-function screen, a promising target worth mentioning—given its actionability in both *KRAS-* and *BRAF*-mutated CRCs—is furin, a member of the proprotein convertase family. This enzyme cleaves many substrates (including growth factors and their receptors, adhesion molecules, angiogenic factors and extracellular matrix proteins) involved in activating multiple tumor-associated signaling pathways dysregulated in CRC, including WNT, NOTCH, MAPK, PI3K and TGFB pathways. It has recently been shown that genetic inactivation of furin suppresses tumorigenic growth, proliferation and migration in mut-*KRAS* or -*BRAF* CRC cell lines, but not in wt-*KRAS* and -*BRAF* cells. In a mouse xenograft model, these mut-*KRAS* or -*BRAF* cells lacking furin displayed reduced growth and angiogenesis, and increased apoptosis. Mechanistically, furin inactivation prevents the processing of various protein precursors, including proIGF1R, proIR, proc-MET, proTGFB1 and NOTCH1, leading to potent and durable ERK–MAPK pathway suppression in *KRAS* or *BRAF* mutant cells [153]. In addition, furin inhibition also improves T-cell targeting of microsatellite instable and stable CRCs via regulation of PD-1 expression, suggesting that its targeting may represent an adjunct approach to colorectal tumor immunotherapy [154].

## 3. CRISPR/Cas9 Knockout Screens

Since the CRISPR/Cas9 system was discovered, it became immediately evident that it was a rapid and powerful tool for gene editing and for phenotypic loss-of-function screening. Instead of targeting mRNAs as in the case of si/shRNA libraries, CRISPR/Cas9 libraries allow us to produce knockout cells by delivering into the cells single-guide (sg) RNAs, whose pairing with specifically targeted sites in DNA trigger the system to make a double-strand cut, whose incorrect repair disrupts gene function.

### Bromodomain and Extra-Terminal Domain (BET) Family

The bromodomain and extra-terminal domain (BET) family of proteins is characterized by the presence of two tandem bromodomains and an extra-terminal domain. Bromodomains specifically bind acetylated lysine residues on the *N*-terminal tails of histones and recruit chromatin-modifying enzymes to target promoters, thus playing a crucial role in regulating gene transcription. Bromodomain-containing protein 4 (BRD4) is required to maintain chromatin stability and controls the transition of cells from M phase to G1 phase during cell cycling, in part, through recruitment of P-TEFb, which, by phosphorylating serine 2 on the carboxyl-terminal domain of RNA Pol II, allows transcription elongation [155].

To uncover the key epigenetic regulators that drive colon cancer growth, McCleland and colleagues developed an arrayed epigenetic CRISPR library targeting the 5′ exons of over 200 genes involved in epigenetic regulation. They evaluated the cell viability of different CRC cell lines 7 days after transduction [156]. Among the 12 top-scoring genes whose knockdown significantly affected colon cancer proliferation, BRD4 was deemed particularly attractive to pursue, given that BRD4 small-molecule inhibitors have entered clinical trials for several hematological malignancies and few reports have studied BRD4 in CRC [157,158]. Two alternatively spliced BRD4 transcripts are expressed: a long-isoform BRD4 (BRD4-LF) and a short-isoform BRD4 (BRD4-SF) [159,160]. McCleland and colleagues found that, whereas overall BRD4 levels remained unchanged at different stages of colonic tumorigenesis, the BRD4-LF isoform, which has been more strongly implicated in transcriptional regulation, was specifically upregulated during the premalignant-to-malignant transition (adenoma to carcinoma) and was highly expressed in CRC cell lines. Upon CRISPR/Cas9 deletion of BDR4, cell lines displayed substantial growth retardation, marked by cell cycle defects consistent with a G1/S-phase delay, which was rescued by BRD4-LF but not BRD4-SF re-expression. In addition, BRD4 constructs containing bromodomain-inactivating mutations failed to rescue the growth defect. In two xenograft models, a doxycycline-inducible shRNA system was used to acutely reduce BRD4 expression after tumors reached 200 mm^3^ in size; in both cases this led to tumor regression characterized by a strong decrease in phosho-histone 3 (proliferation marker) levels and of the direct BRD4 transcriptional target MYC, and no significant change in cleaved caspase-3. In addition, in the HT-29 xenograft model, histopathological analysis of the tumors revealed morphological alterations consistent with cell differentiation and loss of cancer-associated cytological features, suggesting that in vivo BRD4 is required not only for tumor growth but also for maintenance of a dedifferentiated state. Testing the effects of the BET small-molecule inhibitor JQ1 in a panel of 20 colon cancer cell lines with similar proliferation rates, a subset of six cell lines exquisitely sensitive to JQ1, which were CIMP+ was identified, while the six most resistant cell lines were all CIMP-. These findings were confirmed using I-BET-762, an additional BET inhibitor with clinical activity. To identify direct BET-dependent target genes accounting for the increased sensitivity of CIMP+ colon cancer cells, four CIMP+ and two CIMP- CRC cell lines were profiled by RNA-seq, whereas ChIPseq was used to profile the genomic enrichment of lysine 27 acetylation of histone H3 (H3K27ac); this approach uncovered an enrichment of MYC pathway gene signatures in the CIMP+ cells. Accordingly, MYC protein was dramatically reduced in CIMP+ cell lines 24 h after JQ1, and restoration of MYC expression in a BRD4-deficient setting led to partial rescue of cell growth, indicating that that those CIMP+ cells are sensitive to loss of MYC in a BET-dependent manner. The transcriptomic (RNA-seq) and genomic (ChIP-seq) analyses were then integrated to identify genes that were both downregulated after JQ1 treatment and marked by an adjacent superenhancer. Notably, one of the most highly downregulated genes in CIMP+ cells upon JQ1 treatment was the CCAT1 transcript, a lncRNA expressed in colon cancers and reported to regulate MYC expression. Moreover, basal CCAT1 RNA levels correlated with the amount of BRD4 binding and were exquisitely sensitive to JQ1, suggesting that it is a direct BET transcriptional target. Consistent with this superenhancer driving MYC transcription, JQ1 treatment preferentially reduced c-MYC expression in CCAT1-expressing cells; therefore, CCAT1 is a superenhancer template RNA that may serve as a predictive biomarker to identify tumors that utilize BET-mediated MYC transcription for tumor growth. In addition, BET-dependent CCAT1 expression may serve as a pharmacodynamic marker of BET inhibition. Finally, CCAT1 expression was scored in a cohort of normal colon tissues (*n* = 555) and colon tumors (*n* = 705) with associated clinicopathological variables; normal colon tissues showed weak-to-no CCAT1 expression compared with expression levels in CRC. Kaplan–Meier analyses revealed that both overall and CRC-specific 5-year patient survival rates were significantly lower in CCAT1-high tumors compared with survival rates for patients with CCAT1-low tumors. CCAT1 expression correlated with tumor grade (poor differentiation), tumor stage (stages III and IV) and non-mucinous histology, indicating that it is an independent prognostic indicator, able to predict poor survival, independent of cancer stage. The authors then proposed that CCAT1 may serve as a clinical biomarker to predict which cancers utilize BET activity to drive MYC transcription and tumor growth, and to identify patients who are likely to benefit from BET inhibitors.

Targets identified by genetic screens are summarized in Table 2.

## 4. Chemical Screens

The progressive reduction in the cost of automated screening equipment, the availability of different compound libraries and the development of information technology that occurred in the last two decades made it feasible to perform large-scale screenings which promptly stimulated the efforts to identify small-molecule regulators of altered cell signaling associated with cancer development.

### 4.1. BET Inhibitors

Further support to the data discussed in the previous paragraph, about BET proteins being attractive targets in CRC, comes from the recent work from the Shim group, which used a different strategy—i.e., a synthetic lethality drug screening with a library of small-molecule inhibitors targeting the human epigenetic machinery—to uncover BET inhibitors (BET-i) as being synthetic lethal for CRC cells defective for SMAD4 [161]. The high-throughput screening was performed in HCT116 cells rendered SMAD knockout via CRISPR-Cas9 gene editing, and the identified hits were validated both in the same cell line and in another widely used model, such as DLD1, also similarly rendered SMAD knockout. In addition, using HCT116^SMAD+/−^ it was also demonstrated that synthetic lethality of BET-i is dependent on SMAD4 expression level. Several BET-i (OTX-015, I-BET-151, CPI-203, (+)-JQ1 and I-BET-761) scored positively in the validation phase, with OTX-015 being the best candidate. Notably, this inhibitor, clinically known as birabresib, is currently in clinical trials for advanced solid tumors and hematological malignancies. Birabresib was demonstrated to selectively induce G1 cell cycle arrest in SMAD4 knockout cells, via significant reduction in MYC levels and induction of the negative cell cycle regulator p21. Accordingly, ectopic overexpression of MYC or the silencing of p21 could rescue birabresib-induced growth arrest in SMAD4 knockout cells. In in vivo experiments, birabresib significantly reduced the tumor volume of HCT116 ^SMAD4−/−^ xenografts, while it did not affect the growth of HCT116 ^SMAD4+/+^ xenografts, further supporting the synthetic lethal interaction between BET inhibition and SMAD4 loss. In addition, in an in vivo setting birabresib induced synthetic lethality via restoring loss of MYC repression in SMAD4-deficient CRC cells, confirming the data from McCleland and colleagues about the feasibility of treating MYC-overexpressing tumors with BET-i.

### 4.2. Inhibiting the WNT/Beta-Catenin Pathway

Given that dysregulation of beta-catenin-mediated transcription is a key molecular lesion in CRC, it became immediately clear that interrupting this signal would represent a rational and targeted therapeutic approach. First of all, the goal could be achieved via different strategies, such as promoting beta-catenin degradation, stabilizing the destruction complex or antagonizing its binding to the TCF partners. The nature of the process to be targeted offered an easy experimental read-out system to rapidly test for inhibitors in a high-throughput assay, as sensitive and highly scalable as an assay of reporter gene activation. Therefore, since the beginning of the century the search for small-molecule inhibitors of beta-catenin has engaged many efforts from both academic labs and industries.

Lepourcelet and colleagues first developed a binding assay for high-throughput screening using microtiter plates coated with the fragment of beta-catenin that binds to TCF4. Plates were sequentially incubated with a TCF4 fragment (residues 8–54) fused to glutathione-S-transferase (GST), anti-GST antibody and alkaline phosphatase (AP)-conjugated secondary antibody so that the reaction gave a strong AP signal. Then, 7000 purified natural compounds from proprietary and public collections were screened for the ability to inhibit AP signal, and eight of them displayed reproducible dose-dependent inhibition of the TCF4/beta-catenin interaction with an IC_50_ < 10 μM. The inhibitory activity of the compounds was then confirmed biochemically by means of electrophoretic mobility shift assays to demonstrate that the compound was able to displace TCF4 from beta-catenin, followed by independent validation in in vitro and in vivo biological systems using different read-outs, such as c-myc or cyclin D1 expression, cell proliferation and duplication of the Xenopus embryonic dorsal axis. In particular, two of them, CGP049090 and PKF 115–584, appeared to antagonize beta-catenin effects in vivo with limited toxicity [162].

Using a different approach, Emami and colleagues used a report gene activation assay constituted by a luciferase gene under the control of several TCF binding sites expressed in CRC cells with deregulated beta-catenin activity, to screen a secondary structure templated small-molecule library of 5000 compounds for inhibitors of beta-catenin/TCF-mediated transcription. Three closely related compounds were isolated from the initial screen and the most potent one, ICG-001, was selected for further investigation. Using a biotinylated derivative of ICG-001 as an affinity reagent, the target of the compound was found to be the transcriptional coactivator CBP, and it was demonstrated that ICG-001 competes with beta-catenin for CBP binding. At the biological level, ICG-001 showed selective growth inhibitory effects in cancer but not in normal colon cells in vitro; it reduced polyp formation in the Min mouse model of CRC progression and strongly suppressed tumor growth in a xenograft model [163]. The lead compound was further developed and progressed to clinical trials.

The reporter gene activation assay was also used as a read-out by Huang and colleagues in a high-throughput screen that led to the identification of XAV939, a molecule able to interfere with beta-catenin-mediated transcription via increased degradation of the beta-catenin itself. These authors demonstrated that the increase in the activity of the destruction complex was due to increased AXIN stabilization as a consequence of the inhibition of TNKS1 and TNKS2 [8].

The Lum group also used the same read-out of Huang, to screen a ~200,000 compound synthetic chemical library, with the difference that the cells used for the screen were transfected with both the reporter gene and an expression construct encoding WNT3A [164]. By this approach, four compounds were isolated that were able to block the transcriptional response acting by inhibiting WNT production (IWP compounds, 6–9), and five compounds that acted as inhibitors of WNT response (IWR compounds, 1–5). In cultured cells, the IWP compounds were more potent pathway antagonists than those in the strongest class of IWRs (~40 versus ~200 nM, respectively). Using biochemical markers of WNT/beta-catenin pathway activation, it was determined that IWP compounds blocked all WNT-dependent biochemical changes assayed (phosphorylation of the LRP6 and DVL, and beta-catenin accumulation) via acting on Porcupine, a Golgi membrane-bound O-acyltransferase that, by adding a palmitoyl group to WNTs, is essential to their signaling ability. IWR compounds, instead, affected only beta-catenin-mediated transactivation, indicating that they targeted regulatory events downstream of WNT receptors engagement. In fact, they increased AXIN levels by stabilizing it, resulting in elevated levels of beta-catenin phosphorylation, thus primed for degradation. When tested in in vitro systems, IWR compounds induced beta-catenin destruction, even in the absence of normal APC protein function, and mimicked the cell growth effects of beta-catenin siRNA in several cancer cell lines that exhibit differences in growth-dependency on WNT/beta-catenin pathway activity [164].

From the screening of a small-molecule library of 22,000 compounds in the luciferase-reporter system, Lee’s group identified a molecule, MSAB, selectively inhibiting proliferation of WNT-dependent but not WNT-independent CRC cell lines and normal cells. Similarly, MSAB strongly inhibited tumor growth of WNT-dependent but not WNT-independent xenografts. The authors demonstrated that MSAB binds directly to beta-catenin, priming it for proteasomal-mediated degradation via facilitating its ubiquitination [165].

Fang et al. used a more particular approach to identify disruptors of the critical interaction between beta-catenin and the transcription factor TCF4. They in fact used AlphaScreen as a read-out for screening a library containing 16,671 World Drug Index-derived compounds, provided by ChemBioNet and designed by the drug design and modeling group of the Leibniz-Institut für Molekulare Pharmakologie, Berlin, Germany [166]. Briefly, the GST-tagged armadillo repeats domain of human beta-catenin (aa residues 134–668) and the His-tagged *N*-terminal region of human TCF4 (aa 1–79) were bound to two types of different beads that, in case of interaction between the two molecules, and the resulting proximity of the two beads, triggered an energy transfer from one bead to the other, resulting in the production of a chemiluminescent signal. From the primary AlphaScreens, compounds that inhibited interactions by at least 40% at 20 μmol/L were selected and retested in the secondary screen using lower concentrations. Due to its excellent chemical properties and expected plasma membrane permeability, compound LF3 was chosen for further validation. LF3 inhibited WNT/beta-catenin signals in cells with exogenous reporters and in CRC cells with endogenously high WNT activity. LF3 also suppressed features of cancer cells related to WNT signaling, including high cell motility, cell cycle progression and the overexpression of WNT target genes. However, LF3 did not cause cell death or interfere with cadherin-mediated cell–cell adhesion. Remarkably, the self-renewal capacity of cancer stem cells was blocked by LF3 in a concentration-dependent manner. Finally, LF3 reduced tumor growth and induced differentiation in a CRC mouse xenograft model [167].

Accumulating evidence shows that the RAS/ERK pathway strongly interacts with the WNT/beta-catenin pathway during the formation and growth of CRCs [168,169]. In fact, RAS stabilization through aberrant activation of WNT/beta-catenin signaling promotes intestinal tumorigenesis [170]. Based on the high frequency of concurrent APC and KRAS mutations and their strong cooperative interaction, therapies targeting both the WNT/beta-catenin and RAS/ERK pathways would therefore be the ideal treatments for human CRC [171]. On this basis, Cha et al. screened a small-molecule library to identify compounds that destabilized both beta-catenin and RAS proteins via inhibition of the WNT/beta-catenin pathway and identified KY1220 as a lead, which was then functionally improved in its derivative, KYA1797K. Both compounds efficiently destabilized beta-catenin and RAS and reduced the proliferation and transformation of various CRC cells harboring APC and KRAS mutations. AXIN was identified as a direct target for KYA1797K; the binding of the two molecules led to enhanced formation of the beta-catenin destruction complex and induced GSK3B activation, leading to phosphorylation of both beta-catenin and KRAS. Phosphorylated beta-catenin and KRAS then underwent BRTC-mediated ubiquitination and proteasome-dependent degradation. Notably, in vivo, KYA1797K significantly suppressed tumor growth and progression both in mouse xenografts of CRC cells harboring APC and KRAS mutations and in the Apcmin/+/KrasG12DLA2 mouse model [172].

Targets identified by chemical screens are summarized in Table 3.

## 5. Multiplex Screens

Large-scale genomic sequencing projects revealed > 100 mutations in any individual CRC. In the last 20 years, there has been a significant and increasing interest in developing drugs targeting mutated cancer gene products or downstream signaling pathways. However, due to the number of mutations involved and inherent redundancy in intracellular signaling, drugs targeting one mutation or pathway have been either ineffective or have led to rapid resistance. To overcome this issue, Bousquet and colleagues devised a strategy whereby multiple cancer pathways may be simultaneously targeted for drug discovery [176]. Given the role of oncogenic KRAS in cancer initiation and progression and that induction of HIF-1α and HIF-2α is triggered by the majority of mutated oncogenes, or by the loss of essential tumor suppressor genes, the authors generated a series of isogenic HCT116 cell lines defective in either oncogenic KRAS or both HIF-1α and HIF-2α, and subjected them to multiplex genomic, siRNA and high-throughput small-molecule screening. They first performed global gene expression analyses and found that global gene expression affected by mut-*KRAS* showed significant overlap with genes affected by both HIF-1α and HIF-2α, particularly those on the MAPK and PI3K/AKT signaling pathways. To identify druggable gene targets that inhibited both the oncogenic KRAS and overactive HIF pathways, the authors then performed a high-throughput siRNA screen targeting 7784 druggable genes (four siRNA/gene) and compared the different isogenic cell lines for cell viability 96 h after transfection. A total of 176 genes were identified as high-confidence hits, as evaluated by differential cytotoxicity observed with three or four siRNAs, and were grouped into those that affect the mut-*KRAS* pathway only, the HIF-1α/ HIF-2α pathway only or both mut-*KRAS* and HIF-1α/HIF-2α pathways. Ingenuity Pathways Analysis (IPA) revealed a significant overlap between the canonical pathways targeted by siRNAs in wt-*KRAS* and HIF-1α/HIF-2α null cells. Top functions affected included RNA post-transcriptional modification, protein ubiquitination and degradation, cellular assembly and organization, cell cycle, molecular transport and RNA trafficking. Notably, although a significant amount of overlap is rarely seen between synthetic lethal screens, genes in the protein ubiquitination and degradation pathway (see USP39 and PSMD14 discussed above) were already identified as being synthetic lethal with mut-*KRAS*. For proof-of-concept studies, the same in vitro system was then screened with commercially available small-molecule libraries composed of 4720 natural products and other compounds. Several compounds showed up as hits from multiple libraries, serving as an internal validation, and dose−response analysis identified 55 compounds that showed differential cytotoxicity with dose titration to lower concentrations. These compounds exhibited overlap in their mechanisms of action, including calcium channel regulation, DNA metabolism, cardiac glycoside functionality, folic acid biosynthesis, microtubule stability, p53 regulation and protein synthesis regulation. Finally, comparing the results from the small-molecule screen and siRNA library screen, overlaps were found at the level of cell cycle checkpoint regulators and DNA replication, protein ubiquitination, DNA damage response regulators, folic acid biosynthesis and microtubule stability. Two cardiac glycosides targeting the Na+/K+-ATPase and already used for therapy (proscillaridin and peruvoside) were eventually validated; they induced significant downregulation of HIF target genes and were more effective in inducing cytotoxicity in the presence of oncogenic KRAS. Finally, the same isogenic cell lines were used to screen a proprietary library of marine natural products leading to the identification of largazole, already known to act as a potent inhibitor of class I HDAC [177] and as being highly and selectively cytotoxic for mut-*KRAS* with an activated HIF pathway. Notably, lagarzole was already reported as having anticancer activity in CRC cells, both in vitro and in vivo [178].

The identification of genes whose suppression could lead to HIF-1 inhibition and/or finding approved drugs able to inhibit the HIF-1 pathway has been the purpose of the multiplex screening via orthogonal assays described by Hsu et al. [179]. The experimental system consisted of a modified HCT116 cell line. An endogenous Nano Luciferase (NanoLuc) reporter allele was inserted by genome editing downstream of and in frame with the last coding exon of the HIF1A gene, thus allowing the study of protein expression from the endogenous promoter. NanoLuc being a small (<20 kDa), very bright (150-fold > firefly luciferase) glow-type luminescence protein, it allows accurate measurement of even low levels of protein expression using a standard luciferase assay. Therefore, the HIF-1α–NanoLuc assay was used in a high-throughput screening to identify potential HIF-1α inhibitors from the NCATS Pharmaceutical Collection, which contains approximately 2500 clinically-approved and investigational drugs. After 18 h of compound treatment, 305 compounds decreased hypoxia-induced HIF-1α–NanoLuc expression in HCT116 cells, 22 of which have been previously reported as HIF-1 inhibitors. Among them there are anthracyclines, chemotherapeutic agents, anti-metabolic nucleotide analogs, calcium channel blockers, cardiac glycosides, quinolone chemotherapeutics and several pharmacological inhibitors that target the RAS/RAF/MEK/ERK and PI3K/AKT/mTOR signaling pathways. To identify drug targets that are related to the HIF-1 signaling pathway, the reporter cell line was used to screen a 960 siRNA druggable target library under hypoxic conditions, uncovering siRNAs that also, in this case, targeted components of the RAS/RAF/MEK/ERK and PI3K/AKT/mTOR pathways, thus confirming the effectiveness of several target-specific inhibitors [179]. The best hits were then tested for their anti-proliferative activity in the HCT116 cell line and for anti-angiogenic activity in an in vitro coculture assay system, uncovering many clinically-approved and investigational drugs that could be re-purposed as anticancer agents. Notably, also in this screen cardiac glycosides (ouabain and proscillaridin A) were the most potent compounds in affecting HCT116 viability at submicromolar IC50 concentrations; together with the results reported by Bousquet and colleagues, these findings point out Na+/K+-ATPase as a novel therapeutic target in CRC. In addition, another compound identified as very effective at submicromolar IC50 concentration is niclosamide, a drug present in the Model List of Essential Medicine from the World Health Organization, used since the 1960s for tapeworm infection and supposedly acting via the uncoupling of the electron transport chain to ATP synthase at the mitochondria [180,181]. Interestingly, niclosamide also scored as a very potent anti-angiogenic agent when tested in a tube formation assay.

## 6. Drug Re-Purposing

Drug re-purposing or re-positioning indicates when new biological effects for known drugs are identified, leading to recommendations for new therapeutic applications. The time from the discovery of a new drug to the market is usually very long and the process can be risky; in fact, a new promising drug with an excellent performance in preclinical studies may fail due to its toxicity or lack of efficacy in clinical trials. In this context, finding new uses for existing drugs is a strategy that has been gaining attention since safety data and the pharmacokinetic profile of an approved drug are already known and they have already been evaluated in the early stages of clinical trials. Given this perspective, in the last decade several high-throughput screens using libraries of FDA-approved drugs in CRC experimental systems have been performed.

### 6.1. Niclosamide

The first reported screen on CRC cells using libraries of FDA-approved drugs was performed by Chen and colleagues, who aimed at identifying drugs that could block WNT-mediated receptor trafficking, and subsequent WNT signaling [173]. Using as a read-out an imaged-based GFP fluorescence assay monitoring FZD endocytosis, niclosamide was identified as promoting FZD endocytosis and DVL downregulation, with subsequent inhibition of WNT3A-stimulated beta-catenin stabilization and LEF/TCF reporter activity. Niclosamide thus appeared to be a good candidate to negatively modulate WNT/FZD signaling by depleting upstream signaling molecules (i.e., FZD and DVL). The same group subsequently verified its putative anti-tumor effect in CRC in in vitro and in in vivo models. An antiproliferative effect was verified in human CRC cell lines and CRC cells isolated by surgical resection of metastatic disease, regardless of APC mutations. Interestingly, niclosamide at concentrations as low as 2 μmol/L was much stronger at inducing apoptosis of CRC cells than 10 μmol/L oxaliplatin, while being non-toxic for normal cells such as fibroblasts or PBMC. Moreover, in mice implanted with human CRC xenografts, orally administered niclosamide significantly reduced tumor growth without apparent side effects [174]. Another group reported that niclosamide was able also to act downstream of FZD and DVL. In fact, niclosamide disrupted the AXIN–GSK3B complex resulting in the suppression of WNT/Snail-mediated epithelial-mesenchymal transition (EMT), and induced mesenchymal to epithelial reversion at nM concentrations, both in vitro and in vivo [175].

Interestingly, Stein’s group reported the identification of niclosamide as the result of a high-throughput screening of 1280 pharmacologically active compounds aimed at identifying drugs able to suppress the expression of S100A4, a calcium-binding protein implicated in promoting metastasis formation in colon cancer. The screen was performed in HCT116 cells expressing an S100A4 promoter-driven luciferase (LUC) reporter gene construct and the effect of niclosamide on cell migration, invasion, proliferation and colony formation was validated in multiple CRC cell lines in vitro. In vivo imaging of niclosamide-treated mice showed reduced liver metastasis compared to control mice after treatment for 26 days and increased overall survival after discontinuing the treatment [182]. Therefore, it appears that niclosamide might act at different steps of the metastatic process, i.e., by suppressing EMT and by affecting migration.

Niclosamide has also been reported to suppress CSC populations and their self-renewal activities, leading to irreversible disruption of tumor-initiating potential in vivo. In this model the drug has been shown to act at an additional level of the WNT pathway, i.e., by downregulating lymphoid enhancer-binding factor 1 (LEF1) expression, which is critical for regulating stemness via direct regulation of doublecortin-like kinase 1 (DCLK1)-B expression, whose levels correlate with CSCs and poor prognosis in CRC patients. Mechanistically, treatment with niclosamide blocked the transcription of DCLK1-B by interrupting the binding of LEF1 to DCLK1-B promoter, thus resulting in DCLK1-B depletion, which was accompanied by subsequent reduction in survival and increased apoptosis of CSCs. Moreover, combinatorial treatment of niclosamide prevented the increase in stemness in surviving cells following 5-FU treatment in vitro. Finally, niclosamide exerted a potent in vivo anti-tumor effect in both HCT116 and PDX xenografts models and in an AOM/DSS-induced spontaneous CRC model [183].

Finally, as illustrated in the previous paragraph, niclosamide was identified also as an inhibitor of HIF signaling [179]. In addition, Suliman et al. reported that the cytotoxic effect of niclosamide was associated with downregulation of the Notch pathway and upregulation of the tumor suppressor miR-200 family [184]. Interestingly, it was shown that niclosamide exerts neuroprotective effects both in vitro and in vivo by limiting oxaliplatin-induced oxidative stress and neuroinflammation while improving the CRC therapeutic response, thus suggesting that it might be a promising therapeutic adjunct to oxaliplatin chemotherapy [185].

Altogether, these data underscore the possibility of niclosamide being a very promising drug for CRC treatment due to its multitarget effects. In fact, a phase 2 trial is currently undergoing to investigate the safety and efficacy of niclosamide in patients with metastasized CRC progressing under standard therapy (NCT02519582) (EudraCT 2014-005151-20). Additionally, given its acting at different levels on the WNT/beta-catenin pathway, another double-blind, randomized controlled trial is recruiting patients with familial adenomatous polyposis (a condition leading to adenomas and eventual adenocarcinomas in colon and duodenum) to evaluate the chemopreventive effect of niclosamide on polyps formation and progression.

### 6.2. Benzimidazole Antihelmintics. 

Using two established colon cancer cell lines, Nygren and colleagues screened a compound library containing 1600 clinically used drugs with the aim to identify molecules that potentially could be re-positioned for colon cancer therapy [186]. Hits were chosen which were able to reduce by <40% cell survival compared with the control at 10 μM drug concentration on both cell lines. By this approach, 68 hits were retrieved (4.25% hit rate) with known antineoplastic agents (anthracyclines and vinca alkaloids) being the top candidates, followed by cardiac glycosides and antihelminthic benzimidazoles (albendazole, mebendazole, oxibendazole and fenbendazole). Notably, as previously discussed, cardiac glycosides have been retrieved also by two different multiplex screenings aimed at identifying HIF inhibitors [179,187]; their identification using different experimental systems and different approaches strongly point to Na+/K+-ATPase as a novel actionable target in CRC. Curiously, the antihelminthic benzimidazoles identified in this screen have a completely different mechanism of action compared to the anti-helminthic niclosamide discussed in the previous paragraph. In fact, niclosamide act at different levels on the WNT/beta-catenin pathway, whereas benzimidazoles disrupt microtubules by binding to tubulin [188] and impair glucose utilization [189]. In particular, mebendazole (MBZ), the most potent compound of the benzimidazole family, can significantly suppress, in a dose-dependent manner, cell viability in 80% of the CRC cell lines of the NCI60 panel, but not in the three cell models with a non-malignant phenotype. In addition, it also induced a gene expression profile that strongly correlated with nocodazole, a well-known tubulin inhibitor with chemical structure similarity. Furthermore, testing the binding affinity of MBZ at 10 μM against a panel of 97 kinases, it was found that MBZ significantly interacts with several protein kinases, including both wild-type and mutated BRAF. Inhibition of BRAF by MBZ has been confirmed subsequently in a melanoma model, where MBZ was shown to synergize with the MEK inhibitor trametinib to inhibit the growth of BRAFWT-NRASQ61K melanoma cells and xenografts [187]; in addition, MBZ-dependent inhibition of the RAF/MEK/ERK pathway has been demonstrated also in a murine model of hepatocarcinoma [190], pointing out MBZ as a novel BRAF inhibitor. Benzimidazole antihelminthics have been found effective in several cancer models of different origin, either alone or in combination with standard-of-care drugs and shown to exert a cancer cell-specific selectivity with minimal cytotoxicity in normal cells. The antitumor effects of benzimidazole antihelminthics are exerted through a plethora of biological actions, such as inhibition of cell viability, migration, invasion and reduction in colony formation, via induction of G2/M cell cycle arrest, apoptosis and autophagy; in addition, they have been shown also to induce differentiation and senescence, to reduce angiogenesis and to overcome drug resistance by acting on the transporters of the multidrug resistance family utilization; finally, they can also have metabolic effects via impairment of glucose utilization [189]. Antitumor effects of benzimidazoles in in vivo models led to prolonged overall survival and progression-free survival, inhibition of tumor growth and reduction in vessel formation and metastasis, all of which without significant side effects. These results are not unexpected given that this class of drugs is extensively employed both in human and veterinary medicine to control internal parasites and has been used throughout the world since its introduction in the 1960s [189]. Interestingly, a case report refers to a near-complete remission of the metastases in the lungs and lymph nodes and a good partial remission in the liver of a patient with a KRAS-mutated advanced sigmoid colon cancer, treated with MBZ for two months the after failure of two previous lines of therapy [191]. Accordingly, MBZ is currently being tested in clinical trials, especially in the adjuvant setting and in combination with standard-of-care drugs.

Actionable targets identified by orthogonal, multiplex genomic, siRNA, high-throughput small-molecule screening and drug re-purposing are summarized in Table 4.

## 7. Clinical Trials

For several of the targets illustrated in the previous sections, specific inhibitors have been developed and are currently being tested in clinical trials, either alone or in combination with other new and old molecules. For example, onvansertib, a specific inhibitor targeting PLK1, is now in phase 1/2 trial in combination with FOLFIRI and bevacizumab for second-line treatment of mCRC patients with a KRAS mutation (https://clinicaltrials.gov/ct2/show/NCT03829410?term=Onvansertib&draw=2&rank=2; (https://clinicaltrials.gov/ct2/show/NCT04446793?term=Onvansertib&draw=2&rank=4; accessed on 15 April 2021).). A novel inhibitor of GSK3B, 9-ING-41, a synthetic lethal target in a subset of PIK3CA-mutated CRCs [71] and in 5FU-resistant tumors [69], is currently undergoing phase 1 and phase 2 trials as a single agent and in combination with cytotoxic agents, in patients with refractory CRCs (https://clinicaltrials.gov/ct2/show/NCT03678883?term=9-ING-41&draw=2&rank=4; accessed on 15 April 2021). In MLH1 and/or MSH3-deficient CRCs, a very promising target has resulted from PRKDC [92], whose inhibitors AZD7648 and nedisertib are also currently entering early clinical trials (https://clinicaltrials.gov/ct2/show/NCT03907969?term=AZD7648&draw=2&rank=1; accessed on 15 April 2021). Synthetic lethality has been discovered for SHP2 inhibitors in BRAF^V600E^ mutant CRCs [145], eventually translating into a phase 1b study where the allosteric inhibitor TNO155 is being tested in combination with the BRAF inhibitor dabrafenib in patients with advanced/metastatic BRAF^V600E^ CRC (https://clinicaltrials.gov/ct2/show/NCT04294160?term=TNO155&draw=2&rank=5; accessed on 15 April 2021).

VEGFR1 blockade has been shown to be synthetic lethal in CRC cells with APC mutations [59]. Fruquintinib, a specific VEGFR1 inhibitor, is now in phase 2 for metastatic CRC patients who failed second therapy (https://clinicaltrials.gov/ct2/show/NCT01975077?term=VEGFR1+colon&draw=2&rank=12; https://clinicaltrials.gov/ct2/show/NCT01762293?term=VEGFR1+colon&draw=2&rank=13; accessed on 15 April 2021). As previously illustrated, BET inhibitors have been discovered as being synthetic lethal for CRC cells defective for SMAD4 [161]. Notably, BET inhibitors are in phase 1 clinical trial for solid tumors (https://clinicaltrials.gov/ct2/show/NCT04089527?term=bet+refractory&draw=3&rank=13; https://clinicaltrials.gov/ct2/show/study/NCT01987362?term=bet+refractory&draw=2&rank=11; accessed on 15 April 2021). Finally, niclosamide is today in phase 2 trial for patients with metastasized CRC progressing under standard therapy (https://clinicaltrials.gov/ct2/show/NCT02519582; accessed on 15 April 2021).

A summary of the clinical trials described above is shown in Table 5.

## 8. Conclusions

The development of drug-resistance toward classic chemotherapeutic and/or targeted therapy is a major obstacle for curing CRC patients. The development and diffusion of novel high-throughput approaches, both genome-wide and chemical, together with a better understanding of the key molecular pathways involved in CRC onset and progression, have allowed in recent years the identification of several novel actionable targets. Historically, the first pathway being repeatedly targeted using different chemical screens has been the WNT/beta-catenin axis, for which several inhibitors acting on different targets along the pathway have been discovered, few of which have progressed to clinical trials. Examples of the identified compounds are inhibitors of TNKS1 and TNKS2, which increase AXIN stabilization (XAV939) [8], inhibitors of WNT production (IWP compounds, 6–9) [164], drugs blocking WNT-mediated receptor trafficking and subsequent WNT signaling (niclosamide) [173,175], compounds acting as inhibitors of the WNT response (IWR compounds, 1–5) [164], molecules able to displace the interaction of beta-catenin with TCF/LEF (CGP049090, KF 115–584, LF3, ICG-001) [162,163,168] and compounds able to stimulate the ubiquitination, and subsequent degradation, of the beta-catenin itself (MSAB) [166].

However, one of the most fruitful approaches has been to exploit cancer vulnerability by inducing synthetic lethality, both by genetic (siRNA, shRNA and CRISP/Cas9 loss-of-function screens) and chemical (target-directed libraries of small molecules, natural compound libraries and chemical libraries) means.

Given that the high number of mutations involved in the onset and progression of CRC (>100) lead to redundancy in intracellular signaling and tumor adaption—via re-wiring of pathways downstream of the inhibited target—targeting one mutation or pathway is, in the long term, insufficient, since resistance eventually occurs [192]. Experimentally, it has been demonstrated that targeting two proteins along the same pathway or co-targeting two pathways at the same time can significantly improve the therapeutic response. Examples of this approach have been: the screening using a small-molecule library performed in APC/KRAS doubly-mutated CRCs cells, which led to the identification of KYA1797; a small molecule that destabilizes both β-catenin and RAS proteins, thus targeting both the WNT/β-catenin and RAS/ERK pathways [172]; and the multiplexed screens (genomic, siRNA and high-throughput small-molecule screening) performed in KRAS-mutated CRC tumors with an overactivated HIF pathway, which led to the retrieval of several known drugs, among which were glycosides, targeting the Na^+^/K^+^-ATPase, and the antihelminthic niclosamide [176,179], both already used for therapy.

Finally, a note to mention is the re-purposing approach that, by finding new targets/action for drugs already in clinical use, allows us to fast-track old drugs for new kinds of patients. By this approach, the antihelminthic niclosamide has been repeatedly identified by different screens and demonstrated to act at different levels of the WNT/beta-catenin pathway, on the pro-metastatic factor S100A4 and by inhibiting HIF signaling [173,182,185].

On the whole, several new targets have been identified in the last two decades by means of different high-throughput approaches—sometimes combining different approaches together—and several inhibitors rapidly progressed to different phases of clinical trials. With the ever-increasing power of the new omics approaches and the development of more high-throughput technologies, it is expected that many other targets will be identified and validated in a not so far future, thus expanding our weaponry against CRC.

## Figures and Tables

**Table 1:** List of mutant and wild-type *KRAS* cell lines from various cancer types used for the validation of synthetic lethal genes identified by Steckel and colleagues [111].

	Colon	Lung	Pancreas	Ovary	Stomach
***KRAS* mut**	DLD-1 (G13D) SW837 (G12C) LOVO (G13D) T84 (G13D) SW620 (G12V) HCC2998 (146T)	NCI-H23 (G12C) NCI-H727 (G12V) NCI-H358 (G12D) NCI-H460 (Q61H)	CFPAC-1 (G12V) HPAF-II (G12D)	OVCAR-5 (G12V)	AGS (G12D)
***KRAS* wt**	DKO-4 DKS-8 KM12 HT-29 SW48	EKVX NCI-H322M NCI-H520 NCI-H522 NCI-H2170	BXPC3	OVCAR-4 SKOV-3	MKN-45

**Table 2:** Actionable targets identified by genetic screens.

Target	Pathway	Model	Drug(s)	Ref
si/shRNA Screens				
***VEGFR1***	WNT/beta-catenin	VEGFR1 silencing/inhibition is synthetic lethal in cells with APC mutations	VEGFR inhibitors II and III (EMD)	[59]
***GSK3B***	RIPK1-independent, PARP1-dependent necroptosis	5-FU resistance bypassed by addition of a GSK3B inhibitor or GSK3B silencing	LiCL	[69]
	Kinases activated downstream of tyrosine receptor(s)	synthetic lethality in cells treated with a GSK3B inhibitor + silencing of other kinase	CHIR-99021, BIO (Tocris)	[70]
	PI3K/mTOR	GSK3B silencing synthetic lethality in cells with PIK3CA mutation	CHIR-99021, SB216763 (Selleck Chemicals), LiCl	[71]
***p65BTK***	RAS/MAPK	5-FU resistance bypassed by addition of a BTK inhibitor or p65BTK silencing	ibrutinib, spebrutinib	[32]
***PRKCD***	PTEN/AKT	irinotecan resistance bypassed by PRKCD inhibition in PTEN-mutated cells	NU7026 (EMD)	[94]
		sensitization to 5-FU/oxaliplatin by PRKCD silencing	shRNA	[91]
	Homologous recombination–mediated DNA repair	PRKDC silencing synthetic lethal in MSH3-mutated cells	KU-0060648 (Selleck Chemicals)	[92]
		PRKDC inhibition in MLH1 and/or MSH3-deficient cells	KU-0060648 (Selleck Chemicals)	[92]
***PLK1***	Mitosis regulation	PLK1 silencing and inhibition synthetic lethal in mut-*KRAS* cells	shRNA, BI 2536 (Selleck Chemicals)	[102]
		maximal efficiency of PLK1 inhibition vs. chemo- and targeted therapy in colon cancer stem cells	BI 2536 (Selleck Chemicals)	[105]
		silencing synthetic lethality in cells treated with a GSK3B inhibitor	shRNA	[70]
		radiosensitivity increase after 24 h of treatment with a PLK1 inhibitor	BI 2536 (Selleck Chemicals)	[106]
		PLK1 inhibition of synthetic lethality in cells with p21Cip1/CDKN1A loss	siRNA, BI 2536, volasertib (Selleck Chemicals)	[107]
***Proteasome***	Protein degradation	silencing of different proteasome components or proteasomal activity inhibition of synthetic lethality in mut-*KRAS* cells synergy of mut-*KRAS* CRC to proteasome inhibitors by pre-treatment with DNA-damaging drugs cells	shRNA, bortezomib	[111]
		no tumor formation in ApcMin/+ mice treated with subunit LMP7 inhibitor	LMP7-/- mice ONX 0914 (Onyx Pharmaceuticals)	[115]
***USP39***	Protein degradation	USP39 silencing synthetic lethality in cells with KRAS mutations	shRNA	[119]
***PTPN11***	RAS/MAPK	PTPN11 silencing synthetic lethality in resistant mut-*BRAF* cells treated with vemurafenib	shRNA	[145]
***HSPA5***	Unfolded protein response	HSPA5 silencing or inhibition of synthetic lethality in mut-*BRAF* cells	siRNA, HA15	[149]
***SFPQ***	Pre-mRNA splicing	SFPQ silencing synthetic lethality in mut-*BRAF* cells	shRNA	[152]

**Table 3:** Actionable targets identified by chemical screens.

Pathway	Compound	Model	Ref
***Epigenetic modification***	BET family inhibitors	BET inhibitors synthetic lethal in cells defective for SMAD4	[161]
***WNT/beta-catenin***	inhibitors of beta-catenin/TCF4/ interaction	reduction in c-myc or cyclin D1 expression; cell proliferation in vitro; interference with duplication of the Xenopus embryonic dorsal axis in vivo	[162]
		reduction in motility, cell cycle progression and overexpression of WNT target genes in CRC cells with endogenously high WNT activity; blockade of self-renewal capacity of CSC; reduction in tumor growth in a xenograft model	[163]
	inhibitors of beta-catenin/TCF-mediated transcription	growth inhibitory effects in cancer but not in normal colon cells in vitro; reduction in polyp formation in the Min mouse model; suppression of tumor growth in a xenograft model	[164]
	tankyrase inhibitor	antiproliferative effect; increased degradation of beta-catenin due to increased AXIN stabilization	[8]
	WNT inhibitors (inhibitors of WNT production, IWP, and inhibitors of WNT response, IWR)	antiproliferative effect (similar to beta-catenin siRNA) of IWR compounds via increase in beta-catenin destruction, also in APC-defective cells	[164]
	beta-catenin binder (priming it for proteasomal degradation)	selective inhibition of proliferation of WNT-dependent but not WNT-independent cells in vitro; strong inhibition of tumor growth of WNT-dependent but not WNT-independent xenografts	[167]
	AXIN binder (promoter of beta-catenin and KRAS degradation)	suppression of tumor growth and progression in mouse xenografts of CRC cells harboring APC and KRAS mutations and in the Apcmin/+/KrasG12DLA2 mouse model	[172]
	niclosamide	antiproliferative effect in vitro; anti-tumoral effect in vivo; negative modulation of WNT/FZD signaling by depletion of upstream signaling molecules, thus effective also in APC-defective cells.	[173,174,175]

**Table 4:** Actionable targets identified by orthogonal, multiplex genomic, siRNA, high-throughput small-molecule screening and drug re-purposing.

Strategy	Compound	Model	Ref
**Targeting, at the same time, KRAS-mutated cells and overactive HIF**	proscillaridin A, peruvoside (cardiac glycosides targeting the Na+/K+-ATPase) lagarzole (HDAC class I inhibitor)	effective in inducing cytotoxicity in KRAS-mutated cells with overactive HIF pathway	[176]
**Targeting overactive HIF**	ouabain and proscillaridin A (cardiac glycosides targeting the Na+/K+-ATPase) niclosamide (anti-helmintic)	anti-proliferative effect in HCT116 cell line and anti-angiogenic activity in an in vitro coculture assay system	[179]
**Drug re-purposing**	niclosamide	negative modulation of WNT/FZD signaling	[173]
		antiproliferative effect in human CRC cell lines and CRC cells isolated by surgical resection of metastatic disease, regardless of APC mutations; reduction in tumor growth in xenograft models	[174]
		disruption of AXIN-GSK3B complex and suppression of WNT/Snail-mediated EMT	[175]
		anti-clonogenic effect, suppression of cell migration, invasion, proliferation in vitro and anti-metastatic in vivo	[182]
		suppression of self-renewal activities of CSC; prevention of the increase in stemness in surviving cells following 5-FU treatment in vitro	[183]
	mebendazole	Strong suppression of cell viability only in tumor but not normal colon cell lines via microtubules disruption (by binding to tubulin), BRAF inhibition, glucose utilization impairment	[186]

**Table 5:** Clinical trials with drugs targeting hits identified by the approaches described in the review.

Target	Drug and Patient Stratification	Trial Identifier
***PLK1***	Onvansertib in combination with FOLFIRI and bevacizumab for second-line treatment of mCRC patients with a *KRAS* mutation	NCT04446793
***GSK3B***	Cytotoxic agents in combination with 9-ING-41, in patients with refractory cancers	NCT03678883
***PRKDC***	AZD7648 alone and in combination with other anti-cancer agents in patients with advanced cancers	NCT03907969
	Combination of nedisertib with avelumab and radiation therapy for advanced/metastatic solid tumors	NCT04068194
***SHP2***	Allosteric inhibitor TNO155 in combination with the BRAF inhibitor dabrafenib in patients with advanced/metastatic BRAFV600E CRC	NCT04294160
***GRP78***	BOLD-100 in combination with FOLFOX for the treatment of advanced solid tumors	NCT04421820
	Dose escalation study of NKP-1339 to treat advanced solid tumors	NCT01415297
***VEGFR1***	Fruquintinib in metastatic CRC patients who failed 2nd therapy	NCT01975077
	Famitinib in patients with advanced CRC	NCT01762293
***BET***	RO6870810 in patients with advanced solid tumors and and expansion study in patients with selected malignancies	NCT01987362
***WNT/beta-catenin pathway***	Niclosamide in patients with mCRC progressing after therapy	NCT02519582
